# Recurrently deregulated lncRNAs in hepatocellular carcinoma

**DOI:** 10.1038/ncomms14421

**Published:** 2017-02-13

**Authors:** Yang Yang, Lei Chen, Jin Gu, Hanshuo Zhang, Jiapei Yuan, Qiuyu Lian, Guishuai Lv, Siqi Wang, Yang Wu, Yu-Cheng T. Yang, Dongfang Wang, Yang Liu, Jing Tang, Guijuan Luo, Yang Li, Long Hu, Xinbao Sun, Dong Wang, Mingzhou Guo, Qiaoran Xi, Jianzhong Xi, Hongyang Wang, Michael Q. Zhang, Zhi John Lu

**Affiliations:** 1 MOE Key Laboratory of Bioinformatics, Center for Synthetic and Systems Biology, Center for Tsinghua-Peking Joint Center for Life Sciences, School of Life Sciences, Tsinghua University, Beijing 100084, China; 2 International Co-operation Laboratory on Signal Transduction, Eastern Hepatobiliary Surgery Institute, Second Military Medical University, Shanghai 200438, China; 3 National Center for Liver Cancer, Shanghai 201805, China; 4 Bioinformatics Division, TNLIST and Department of Automation, Tsinghua University, Beijing 100084, China; 5 Department of Biomedical Engineering, College of Engineering, Peking University, Beijing 100871, China; 6 Department of Neurosurgery, Wuhan General Hospital of Guangzhou Command, Wuhan Hubei 430070, China; 7 Department of Gastroenterology & Hepatology, Chinese PLA General Hospital, #28 Fuxing Road, Beijing 100853, China; 8 Department of Biological Sciences, Center for Systems Biology, The University of Texas at Dallas, 800 West Campbell Road, RL11 Richardson, Texas 75080-3021, USA

## Abstract

Hepatocellular carcinoma (HCC) cells often invade the portal venous system and subsequently develop into portal vein tumour thrombosis (PVTT). Long noncoding RNAs (lncRNAs) have been associated with HCC, but a comprehensive analysis of their specific association with HCC metastasis has not been conducted. Here, by analysing 60 clinical samples' RNA-seq data from 20 HCC patients, we have identified and characterized 8,603 candidate lncRNAs. The expression patterns of 917 recurrently deregulated lncRNAs are correlated with clinical data in a TCGA cohort and published liver cancer data. Matched array data from the 60 samples show that copy number variations (CNVs) and alterations in DNA methylation contribute to the observed recurrent deregulation of 235 lncRNAs. Many recurrently deregulated lncRNAs are enriched in co-expressed clusters of genes related to cell adhesion, immune response and metabolic processes. Candidate lncRNAs related to metastasis, such as *HAND2-AS1*, were further validated using RNAi-based loss-of-function assays. Thus, we provide a valuable resource of functional lncRNAs and biomarkers associated with HCC tumorigenesis and metastasis.

## Abstract (web summary)

Long noncoding-RNAs have been linked to hepatocellular carcinoma (HCC) and some can be used as prognostic markers. Here the authors, by analysing RNA-seq in 60 clinical samples from 20 patients, provide a resource of functional lncRNAs and biomarkers associated with HCC tumorigenesis and metastasis.

Hepatocellular carcinoma (HCC) is one of the most common and aggressive human malignancies^1^. The dismal clinical outcome of HCC is largely due to the high incidence of intrahepatic and extrahepatic metastasis in HCC patients^2^. HCC cells are highly likely to develop into portal vein tumour thrombosis (PVTT), which is the main route for intra-hepatic metastasis of HCC (ref. 3). Therefore, PVTT is closely associated with poor prognosis for HCC patients^4^.

Several long noncoding RNAs (lncRNAs), including *H19* (ref. 5), *HOTAIR* (ref. 6) and *HULC* (ref. 7), are directly involved in tumorigenesis and metastasis of various types of cancer. Recent studies have also revealed the pro-metastasis mechanisms through which some lncRNAs contribute to the activation of epithelial-to-mesenchymal transition networks, including activation of the WNT (ref. 8) and TGF-β signalling pathways^9^. Although several studies have assessed the contributions of individual lncRNAs to the development of HCC, the functions and mechanisms of only a few lncRNAs in HCC tumorigenesis and metastasis are understood in detail^10^,^11^.

Moreover, efforts at systematic identification and characterization of candidate lncRNAs involved in HCC, especially those involved in HCC metastasis, remain at an early stage. A recent study based on The Cancer Genome Atlas (TCGA, http://cancergenome.nih.gov/) revealed the existence of more than 50,000 lncRNAs (designated MiTranscriptome lncRNAs) in the human transcriptome (generated from various tumours, normal tissues and cell lines)^12^, of which more than 80% were not reported in previous studies or found in databases (that is, GENCODE (ref. 13) and Refseq (ref. 14)). This study demonstrates the genomic diversity and expression specificity of lncRNAs, while suggesting that more lncRNAs will be discovered as additional tumour and cell types (for example, metastatic samples) are sequenced.

Remarkably, studies suggest that approximately 88% of single-nucleotide polymorphisms (SNPs) in the human genome are within noncoding regions^15^, suggesting that many noncoding RNAs and DNA regulatory elements (for example, promoters and enhancers) have functional roles. Indeed, some lncRNAs play important roles in diverse cellular process, such as cell differentiation^16^, cell death and tumorigenesis^17^. In addition, lncRNAs can be used as biomarkers for cancer diagnosis, prognosis and classification because they have cell-type specificity better than that of most protein-coding genes and relatively stable local secondary structures, facilitating their detection in body fluids^18^,^19^,^20^. For instance, lncRNA *PVT1* has been used as a diagnostic and prognostic biomarker for HCC (ref. 11).

Here, 60 matched samples (primary tumour, PVTT and adjacent normal tissue) from 20 Chinese HCC patients were subjected to total RNA-seq (rRNA depleted), followed by integrative analysis at the genomic, transcriptomic and epigenomic levels, with the goal of identifying and characterizing deregulated lncRNAs in HCC patients. Approximately 76% of the lncRNAs identified in the samples were not annotated by the MiTranscriptome^12^ or GENCODE transcriptome^13^ databases. Next, approximately 1,000 lncRNAs that were recurrently deregulated in primary tumours and/or PVTTs were identified. Their expression levels were correlated with TCGA clinical data and additional published liver cancer data^21^. We also showed that one of the recurrently deregulated lncRNAs was suitable as a prognosis and metastasis biomarker in HCC patients. Furthermore, copy number variations (CNVs) and DNA methylation alterations were shown to be responsible for the aberrant expression patterns of 147 and 93 recurrently deregulated lncRNAs, respectively. Finally, a coding-noncoding co-expression network was used to predict candidate lncRNAs related to metastasis, after which the predictions were validated experimentally.

## Results

### Identification of candidate lncRNAs in HCC clinical samples

To systematically identify lncRNAs related to HCC tumorigenesis and metastasis, approximately 9.6 billion reads for 60 samples from 20 HCC patients were sequenced using total RNA-seq (rRNA depleted) (Supplementary Data 1). Three matched samples were collected from each patient: primary tumour, adjacent normal tissue and PVTT.

**Figure 1:**
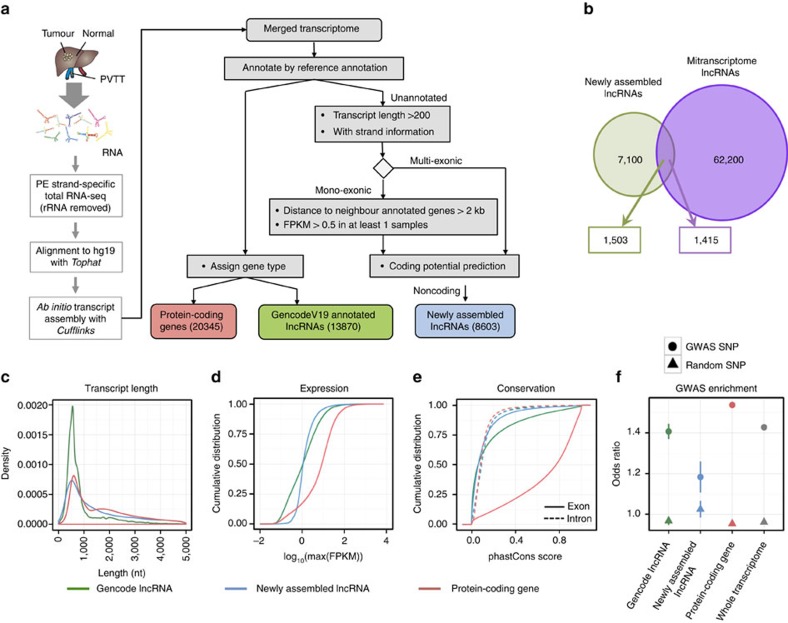
Identification of candidate lncRNAs in 60 HCC samples. (**a**) Overview of the comprehensive experimental and computational scheme for the systematic identification of lncRNAs in samples from HCC patients. (**b**) Venn diagram showing the overlap between newly assembled lncRNAs and MiTranscriptome lncRNAs. Characterization of GENCODE lncRNAs, newly assembled lncRNAs and protein-coding genes: (**c**) transcript length distribution; (**d**) cumulative distribution curve of maximum gene expression level (RPKM); (**e**) conservation of exons and introns; (**f**) enrichment of GWAS SNPs (circle) over randomly selected SNPs (triangle).

We first found that 95.1% of 13,870 lncRNAs (including 23,898 transcripts) annotated by GENCODE (V19) (ref. 13) were detected (FPKM>0.5 for single-exon lncRNAs and FPKM>0 for multi-exon lncRNAs) in our samples, indicating that our sequencing depth was good. Next, using these GENCODE lncRNAs as a reference annotation, we assembled 8,603 candidate lncRNAs (including 10,196 transcripts) (Supplementary Data 2) with a bioinformatics pipeline (Fig. 1a and Supplementary Table 1): (1) assembling RNA transcripts from RNA-seq reads; (2) filtering potential noise based on genomic location, length and expression level; (3) removing transcripts with coding potential, which was calculated by two computational tools, CPC (ref. 22) and COME (ref. 23) (see details in Methods). These candidate lncRNAs were designated as newly assembled lncRNAs. Only a small number of the newly assembled lncRNAs were reported in other studies. For example, 76% of them were not found in the MiTranscriptome database^12^, which was mainly derived from TCGA data (Fig. 1b). The newly assembled lncRNAs were also compared with two other representative annotation databases: a high quality set, RefSeq (ref. 14), and a comprehensive set, NONCODE (over 50,000 lncRNA transcripts included)^24^. Only 2% and 10% of the newly assembled lncRNAs were found in the RefSeq (Release 72) and NONCODE (V4) databases, respectively (Supplementary Fig. 1). These results indicate the depth of our sequencing data and expression specificity of the lncRNAs identified in our samples. We showed that the number of lncRNAs increased when the number of sequenced samples was increased (Supplementary Fig. 2), while the detection ability for protein-coding genes and GENCODE lncRNAs was saturated at approximately 10 and 20 out of 60 samples, respectively.

### Characterization of the candidate lncRNAs

We characterized genomic location, expression abundance, transcript length, conservation and SNP enrichment for the newly assembled lncRNAs (Fig. 1c–f, Supplementary Data 3 and 4). We first focused on the genomic locations of newly assembled lncRNAs (Supplementary Fig. 3A). The majority (74%) of the lncRNAs were located in intergenic regions; 16% were antisense to protein-coding genes, whereas 3% were located in the introns of protein-coding genes. Moreover, 1.39% and 24.94% of the lncRNAs overlapped with pseudogenes and transposable elements, respectively, whereas 2.24% and 5.06% of the lncRNAs contained local domains conserved with canonical ncRNAs (for example, rRNA, tRNA and snRNA) at the sequence and structure levels, respectively. Similarly, the GENCODE lncRNAs also overlapped with or included these elements (Supplementary Fig. 3b,c).

Next, we characterized the basic features of the newly assembled lncRNAs and compared them with protein-coding genes and GENCODE lncRNAs. Because they had fewer putative exons, we found that the newly assembled lncRNA transcripts were shorter than protein-coding genes, but longer than GENCODE lncRNAs (Fig. 1c, Supplementary Fig. 4). This result indicates that the high sequencing depth of our analysis (Supplementary Data 1) enabled us to assemble long transcripts close to their full length, even though they were expressed at low levels (Fig. 1d, Supplementary Data 4 and 5). Notably, the newly assembled lncRNAs were less evolutionarily conserved in comparison with protein-coding genes and GENCODE lncRNAs, while exonic regions were more conserved in comparison with intronic regions (Fig. 1e).

A previous study reported that almost 90% of SNPs are located in non-coding regions^15^. To investigate the relationship between lncRNAs and diseases, we capitalized on the GWAS SNP Catalog from NHGRI (ref. 25). We intersected the lncRNAs with GWAS SNPs from the NHGRI catalogue and randomly selected SNPs from the dbSNP (ref. 26). Interestingly, we found that GWAS SNPs were significantly enriched in the newly assembled lncRNAs in comparison with a random set (Fig. 1f). These data suggested that our newly assembled lncRNAs were likely to be functionally associated with human diseases.

To further validate the activity of the newly assembled lncRNAs, we used published ChIP-seq data for the HepG2 cell line^27^ to depict activity markers around transcription start sites (TSSs). Different epigenetic signatures (H3K4me3, H3K27ac, Pol II binding, DNase I hypersensitivity) indicated active transcription of the newly assembled lncRNAs in liver cancer cell lines (Supplementary Fig. 5). Peaks of these markers were found at the TSSs of lncRNAs, suggesting that the promoters of these transcripts are actively regulated in HepG2 cells.

### Recurrently deregulated lncRNAs in tumours and PVTTs

**Figure 2:**
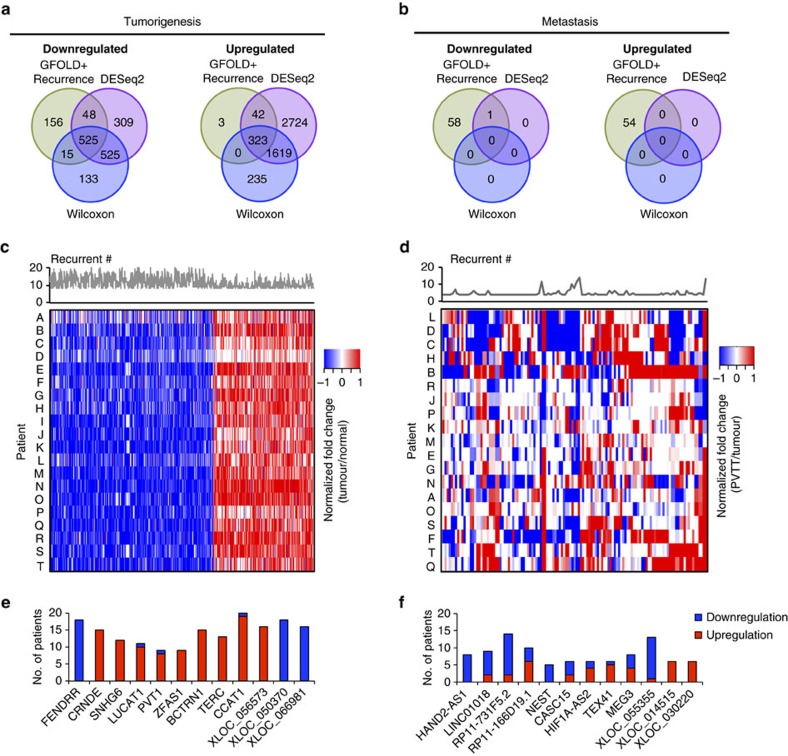
Recurrently deregulated lncRNAs in primary tumours and PVTTs. Identification of recurrently deregulated lncRNAs: recurrently downregulated and upregulated lncRNAs that were predicted by three statistical methods to be associated with (**a**) tumorigenesis and (**b**) metastasis. Fold-change of expression in each individual patient for (**c**) recurrently deregulated tumorigenesis-associated lncRNAs; (**d**) recurrently deregulated metastasis-associated lncRNAs (tumour versus PVTT). Patient I was not included in the analyses related to metastasis because the PVTT sample of patient I was contaminated. Stacked bar charts showing examples of recurrently deregulated lncRNAs, including tumorigenesis-associated (**e**) and metastasis-associated (**f**) lncRNAs. The number on the *y* axis is the number of patients with differential expression of each lncRNA.

We used three statistical methods, GFOLD (ref. 28) followed by counting recurrences in multiple patients (Supplementary Figs 6–7), DESeq2 (ref. 29) and Wilcoxon signed-rank test to define lncRNAs (including both GENCODE lncRNAs and newly assembled lncRNAs) that were differentially expressed recurrently (Fig. 2a,b, Supplementary Data 6) (see details in Methods).

For the comparison of primary tumours and adjacent normal tissues, we found that the results of DESeq2 (ref. 29) and Wilcoxon signed-rank test were more similar to each other than to the results of GFOLD (Fig. 2a), because the former two methods both treated the patients as replicates, whereas GFOLD assessed differential expression based on individual patients. Finally, we identified 525 and 323 lncRNAs as recurrently downregulated or upregulated, respectively, in primary tumours by overlapping the predictions of all three methods (Fig. 2a,c). The identified lncRNAs were considered to represent a group of recurrently deregulated lncRNAs potentially associated with tumorigenesis. These lncRNAs and their *P* values, q-values and expression fold-changes are listed in Supplementary Data 6.

For the comparison of PVTTs and primary tumours, we found that DESeq2 and Wilcoxon signed-rank test identified very few differentially expressed lncRNAs (Fig. 2b), because of the relatively high heterogeneity of PVTTs (Fig. 2d). Only one lncRNA (*HAND2-AS1*) was identified as a downregulated candidate in PVTTs. Paired primary tumours and PVTTs from individual patients (average correlation coefficient: 0.99) were more similar than PVTTs from different patients (average correlation coefficient: 0.76). Therefore, we used GFOLD to evaluate differential expression by treating patients individually, followed by counting recurrences in multiple patients (see Methods), revealing 107 lncRNAs that were defined as recurrently deregulated lncRNAs potentially associated with metastasis (Fig. 2d, Supplementary Data 6). Notably, of the 107 metastasis-associated candidates, 38 were also recurrently deregulated in primary tumours in comparison with adjacent normal tissues (see details in Discussion).

Examples of tumorigenesis-associated lncRNAs are shown in Fig. 2e. Some of the tumorigenesis-associated lncRNAs identified in this study have been reported by previous studies. For instance, *PVT1* promotes cell proliferation, cell cycle progression and development of stem cell-like properties in HCC (ref. 11). In addition, several lncRNAs (for example, *TEX41*, *XLOC_014515* and *XLOC_030220*) were recurrently upregulated in PVTT samples, whereas others (for example, *HAND2-AS1*, *RP11-731F5.2* and *XLOC_055355*) were recurrently downregulated in PVTTs (Fig. 2f). For example, *HIF1A-AS2*, a lncRNA antisense to hypoxia-inducible factor 1-alpha, is overexpressed in gastric cancer cells and involved in gastric cancer development^30^. Notably, we identified some newly assembled lncRNA candidates potentially related to metastasis, including *XLOC_014515* and *XLOC_030220* (Supplementary Data 6).

### Association of deregulated lncRNAs with public clinical data

**Figure 3:**
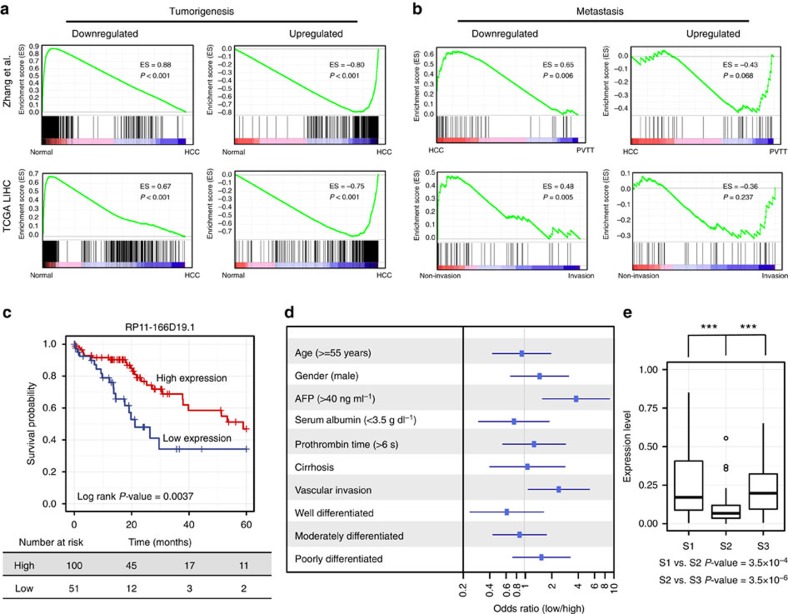
Association of recurrently deregulated lncRNAs with TCGA clinical data and other published data. (**a**) Gene set enrichment analysis (GSEA) of recurrently deregulated tumorigenesis-associated lncRNAs based on TCGA LIHC data and published liver cancer data. lncRNAs were rank-ordered by differential expression between adjacent normal tissue and primary tumour samples. (**b**) GSEA of recurrently deregulated metastasis-associated lncRNAs. lncRNAs were rank-ordered by differential expression between primary tumours with and without vascular invasion in the TCGA LIHC data, as well as by differential expression between primary tumours and PVTTs in published liver cancer data. (**c**) Kaplan-Meier analysis of overall survival in the TCGA LIHC cohort. Subjects were stratified according to the expression of lncRNA RP11-166D19.1. The *P* value for Kaplan-Meier analysis was determined using log-rank test. (**d**) Multivariate analysis using additional clinical information. Forest plot depicting correlations between the indicated clinical criteria and the expression level of *RP11-166D19.1*. (**e**) Expression levels (TCGA data) of *RP11-166D19.1* in three HCC subclasses (S1, S2 and S3). ***P value<0.001, Wilcoxon rank-sum test.

Based on Gene Set Enrichment Analysis (GSEA) (see Methods) of recurrently deregulated lncRNAs, we found that the expression levels of the tumorigenesis- and metastasis-associated lncRNA sets were consistent with their expression levels in another published data set from 11 matched normal tissue samples, primary tumours and PVTTs (ref. 21) (Fig. 3a,b). In addition, we explored the expression levels of recurrently deregulated lncRNAs in a TCGA liver hepatocellular carcinoma (LIHC) cohort (see Methods). Tumorigenesis-associated lncRNAs were also aberrantly expressed between normal tissues and primary tumours. Because the TCGA LIHC cohort had no PVTT or metastatic tumour samples, we compared expression levels of metastasis-associated lncRNAs between primary tumours with and without invasion, revealing that deregulation of metastasis-associated lncRNAs was in accord with the clinical status of the TCGA patients (Fig. 3a,b).

The consistent expression levels of recurrently deregulated lncRNAs in our samples and the TCGA LIHC cohort suggest that the identified lncRNAs could potentially serve as biomarkers. As an example, we explored a metastasis-associated lncRNA, *RP11-166D19.1* (ENSEMBL ID: *ENSG00000255248*, an isoform of *MIR100HG*), which was also significantly downregulated in 4 of 11 PVTTs in comparison with matched primary liver tumours in a previous study^21^. We found that *RP11-166D19.1* could be used to clearly classify the patients in the TCGA cohort into two subclasses with different survival rates. *RP11-166D19.1* expression was significantly associated with the overall survival time of HCC patients' (log rank *P* value=0.0037) (Fig. 3c). Moreover, a multivariate analysis based on additional clinical information showed that the HCCs of the low *RP11-166D19.1* expression subclass were globally more severe than those of the high-expression subclass: the low expression subclass was significantly more likely to have AFP≥40 ng ml^−1^, had a high risk of vascular invasion, and had a clear tendency to have serum albumin<3.5 g dl^−1^ and prothrombin time>6 s. The patients in the high-expression subclass were mostly well-differentiated (Fig. 3d).

Three HCC subclasses (S1, S2 and S3) were identified in a previous HCC study^31^,^32^. Here, we clustered 20 primary tumours from our study, as well as 157 TCGA LIHC tumours and 11 HCC tumours from a previous study^21^, into three subclasses (Supplementary Fig. 9) based on the expression patterns of 619 signature genes^31^. We also identified lncRNAs that were significantly deregulated in each subclass (Supplementary Fig. 10, Supplementary Data 7). Interestingly, the putative metastasis biomarker *RP11-166D19.1* was found to be significantly downregulated in subclass S2 in comparison with its expression level in subclasses S1 and S3 (Wilcoxon rank-sum test, *P* value=3.5 × 10^−4^ and *P* value=3.5 × 10^−6^, respectively) (Fig. 3e).

### DNA methylation alternations and CNVs of lncRNAs

**Figure 4:**
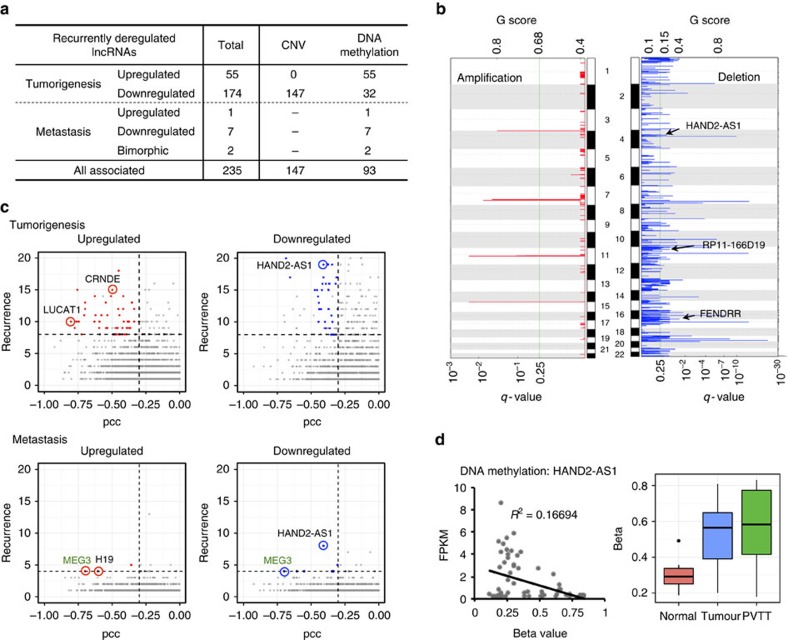
Regulatory mechanisms for recurrently deregulated lncRNAs. (**a**) Summary of the regulatory mechanisms of recurrently deregulated lncRNAs. The numbers of lncRNAs associated with CNV and/or DNA methylation data are listed. Bimorphic lncRNAs were those that were recurrently upregulated in some patients and recurrently downregulated in other patients. The total number of lncRNAs is not equal to the sum of each type because of overlapping sub-types. (**b**) Chromosomal view of amplification and deletion peaks between primary tumours and normal tissue. The G-scores (top) and FDR q-values (bottom) of peaks were calculated using GISTIC2.0. The G-score considered the amplitude of the aberration and its frequency of occurrence across all samples. The q-value was calculated for the observed gain/loss at each locus using randomly permuted events as a control. Examples of recurrently deregulated lncRNAs located in the peaks (only found in deletions) are labelled. (**c**) Scatterplots showing recurrently deregulated lncRNAs (colour labelled) that were putatively affected by alterations in DNA methylation. Recurrently deregulated lncRNAs driven by DNA methylation had expression levels that were inversely correlated with DNA methylation levels at their promoter regions (PCC, Pearson correlation coefficients<−0.3, *x* axis). (**d**) Example of a recurrently deregulated lncRNA driven by DNA methylation; the *HAND2-AS1* expression level (FPKM) was inversely correlated with its promoter methylation level (beta value). Boxplot showing that the beta values of primary tumour samples were significantly higher than those of normal tissue samples, but slightly lower than those of PVTT samples.

We categorized the recurrently deregulated lncRNAs based on their correlations with CNVs and/or DNA methylation alterations assayed in our matched samples (Fig. 4a). We listed all recurrently deregulated lncRNAs (Supplementary Data 8) with expression patterns correlated with CNV data (that is, upregulated lncRNAs were found to be located in a DNA amplification region) and/or DNA methylation data (that is, downregulated lncRNAs had strong DNA methylation signals at their promoter regions). Several lncRNAs were recurrently upregulated in some patients and recurrently downregulated in other patients; such lncRNAs were designated as bimorphic lncRNAs (Fig. 4a).

The CNVs of recurrently deregulated lncRNAs in HCC cells were analysed using CytoscanHD arrays. Based on a GISTIC analysis^33^, 4 significantly amplified genome regions and 70 significantly deleted genome regions were revealed in our samples (Fig. 4b). To characterize candidate CNV-driven lncRNAs, we mapped recurrently deregulated lncRNAs to amplified and deleted genome regions. In total, 147 recurrently deregulated lncRNAs were identified in deleted regions, whereas none were found in amplified regions (Fig. 4a, Supplementary Data 8). For example, *FENDRR*, which was reported to be a prognostic biomarker in gastric cancer^34^, had a pattern of decreased expression driven by copy number deletion (Fig. 4b).

Based on DNA methylation microarrays of 60 matched samples, we analysed DNA methylation patterns to identify recurrently deregulated lncRNAs that were affected by alterations in DNA methylation. We applied several separate filtering criteria (see Methods) to define recurrently deregulated lncRNAs driven by alterations in DNA methylation. In total, 93 (10.1%) recurrently deregulated lncRNAs had significant correlations between DNA methylation and expression levels (Fig. 4a,c, Supplementary Data 8), suggesting that their expression levels in tumour and/or PVTT samples were probably regulated by DNA methylation. As an example, we showed that the expression level of a recurrently deregulated lncRNA, *HAND2-AS1*, was inversely correlated with the DNA methylation level at its promoter region (*R*^2^=0.16694) (Fig. 4d). The promoter region of *HAND2-AS1* was hypermethylated in primary tumours and PVTT samples.

### Inference of lncRNA function using a co-expression network

**Figure 5:**
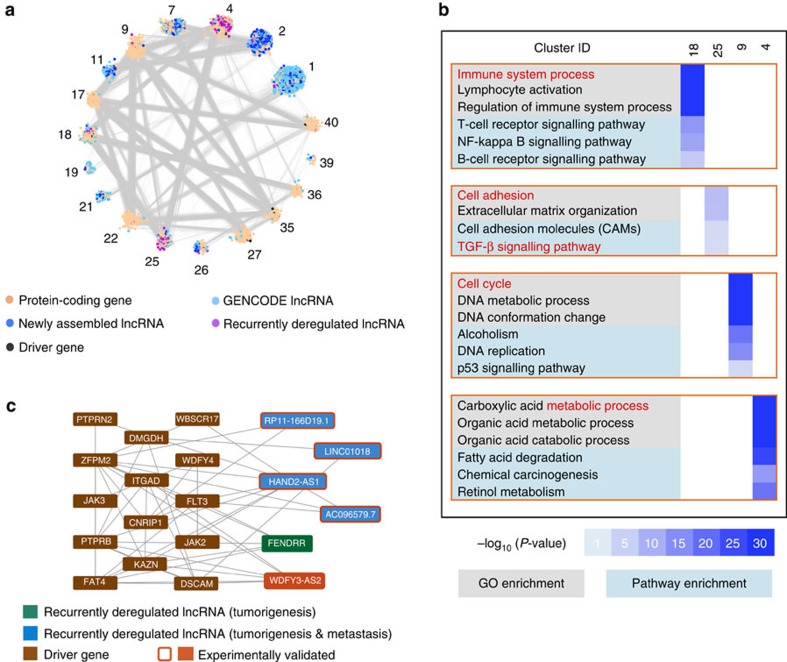
Inference of potential functions for recurrently deregulated lncRNAs using a co-expression network. (**a**) Network representation of 18 selected inter-connected clusters in the coding-non-coding co-expression network. (**b**) GO and KEGG pathway enrichment for four selected clusters (4, 9, 18 and 25). Heatmap showing enrichment scores (−log_10_(*P* value)) for GO terms and KEGG pathways in four selected clusters. The most significantly enriched GO terms and KEGG pathways are displayed. (**c**) Sub-network showing important genes/lncRNAs in cluster 25. The subnetwork depicts the relationships among four lncRNAs and liver cancer-related driver genes.

To predict the potential functional and regulatory mechanisms of lncRNAs with respect to the molecular aetiology of HCC, we constructed a co-expression network^35^ of protein-coding genes and lncRNAs (see Methods). The resulting co-expression network consisted of 7,367 protein-coding genes, 6,377 GENCODE lncRNAs and 5,612 newly assembled lncRNAs. There were 1,286 recurrently deregulated lncRNAs in the network. All protein-coding genes and lncRNAs were grouped into 43 clusters, each of which had at least 100 highly inter-connected genes (Supplementary Data 9). In addition to interaction edges within a cluster, there were also 337,609 edges between nodes in different clusters, which could indicate their functional relatedness or regulatory relationships (Fig. 5a, Supplementary Fig. 11).

Among the 43 clusters, we found four clusters containing protein-coding genes with interesting functions: clusters 4, 9, 18 and 25 (Fig. 5b). The recurrently deregulated lncRNAs are highly enriched in these gene clusters (Fisher's exact test, all *P* values<0.01). For example, Gene Ontology and KEGG pathway enrichment analyses suggest that the protein-coding genes in cluster 4 are mostly associated with metabolic processes in the liver, such as organic acid metabolism and degradation of fatty acids. The protein-coding genes in cluster 9 mainly function in cell cycle processes such as DNA repair, DNA replication and DNA metabolism, which influence cell migration^36^. Cluster 18 is enriched with immune response genes involved in the T-cell and B-cell receptor signalling pathways, consistent with reports that immune and inflammatory responses play decisive roles in tumour development by influencing the processes of invasion and migration^37^.

Another intriguing example is cluster 25 (Fig. 5c), which includes protein-coding genes enriched in functional terms related to metastasis, such as cell adhesion and the TGF-β signalling pathway, which have been shown to play essential roles in diverse processes, including cell proliferation, differentiation, motility and adhesion^38^. Furthermore, many HCC-related driver genes^39^ are also found in cluster 25. For example, the *FLT3* receptor plays a role during the late stages of liver regeneration and is involved in GPCR signalling pathways^40^. *FLT3* was co-expressed with other driver genes in the sub-network, including *WDFY4* and *FAT4*. Moreover, three recurrently deregulated lncRNAs (*HAND2-AS1*, *AC096579.7* and *FENDRR*) were strongly correlated with *FLT3* at the expression level, suggesting that these lncRNAs have functions related to that of *FLT3*. Another interesting gene in cluster 25, *FAT4*, encodes a cadherin (a calcium-dependent cell adhesion protein) that serves as a tumour-suppressor gene^41^. *FAT4* was closely associated with some recurrently deregulated lncRNAs, including *HAND2-AS1*, *FENDRR* and *WDFY3-AS2*, all of which were differentially expressed during cell migration. Cell adhesion was one of the most significantly enriched processes during tumour metastasis. These co-expression relationships provide functional evidence demonstrating that adhesion-related lncRNAs likely have roles in tumour metastasis. Furthermore, driver gene *ZFPM2* was also highly involved in the sub-network; it was significantly correlated with seven driver genes and five recurrently deregulated lncRNAs. Some migration-related recurrently deregulated lncRNAs (*HAND2-AS1*, *AC096579.7* and *FENDRR*) had expression patterns similar to that of *PTPRB*, which plays an important role in blood vessel remodelling and angiogenesis^42^, indicating that these lncRNAs could have related functions.

### Functional assay for metastasis-related lncRNA candidates

**Figure 6:**
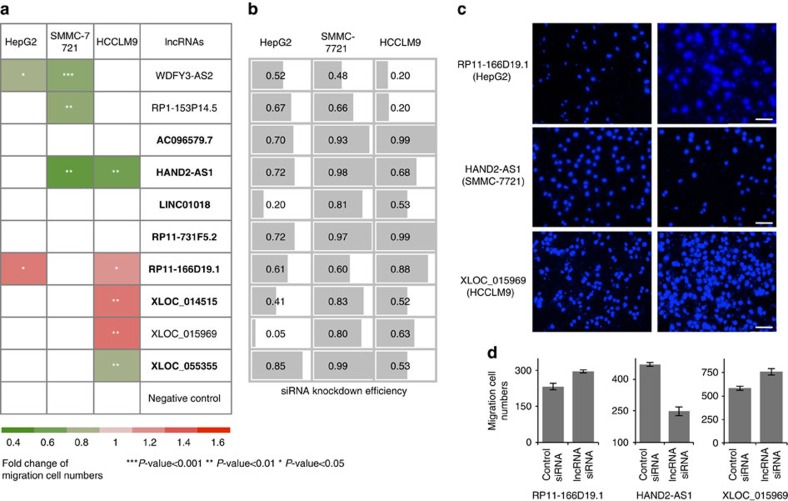
Loss-of-function assay of candidate lncRNAs regulating cell migration. Transwell migration assays were conducted to test the effects of siRNA-mediated RNAi of candidate lncRNAs in three liver cancer cell lines: HepG2, SMMC-7721 and HCCLM9. (**a**) The value in the heatmap is the fold-change (*P* value<0.05) of the transwell cell numbers for knockdown cells over those of control cells. All results are expressed as the mean derived from three independent experiments. The unpaired Student's *t*-test (two-tailed) was used for comparisons of two groups. Seven of the ten candidate lncRNAs were metastasis-associated lncRNAs that were recurrently deregulated in PVTTs (in bold font). *P value<0.05, **P value<0.01, ***P value<0.001, *t*-test, *n*=3. (**b**) RNAi was validated by qRT-PCR. Examples of migration phenotype: transwell cells (DAPI staining) (**c**) (Scale bar, 500 μm) and their counts (**d**). Error bars represent the s.d. of three experiment replicates.

Transwell migration assays were used to assess whether putative candidate lncRNAs might function in the progression of HCC. We first selected ten lncRNA candidates that were associated with cell adhesion based on the co-expression network described above (Supplementary Table 2), in which four lncRNAs (*WDFY3-AS2*, *HAND2-AS1*, *RP11-166D19.1* and *XLOC_055355*) were also significantly co-expressed with genes in the TGF-β signalling pathway. Note that seven candidate lncRNAs (shown in Fig. 6a) were recurrently deregulated lncRNAs in PVTT samples and designated as metastasis-associated lncRNAs in the sections above. Three siRNAs were synthesized for each candidate lncRNA and mixed as a pool (Supplementary Table 3). Three liver cancer cell lines, HepG2, SMMC-7721 and HCCLM9, were used to conduct loss-of-function RNAi assays (Fig. 6a).

Remarkably, knockdown of seven of the ten candidate lncRNAs significantly affected cell migration in at least one cell line; knockdown of three lncRNAs produced accordant alterations in cell migration in at least two cell lines by suppressing or promoting cell migration (Fig. 6a, Supplementary Table 3). RNAi knockdown efficiency was confirmed using qRT-PCR (Fig. 6b, Supplementary Fig. 12, Supplementary Table 4). The results from three representative transwell assays are shown on the right side of the figure (Fig. 6c,d). In order to document the reproducibility of the results, we repeated the RNAi knockdown and transwell migration assays using three siRNAs separately in all three cell lines (Supplementary Fig. 13A–B and Supplementary Table 5); the results of these experiments were consistent with those of the mixed siRNA experiments. Moreover, to avoid mistaking differences in cell proliferation for differences in cell migration, we performed CCK8 cell proliferation assays, revealing that knockdown of these lncRNAs hardly affected cell proliferation (Supplementary Fig. 13C, Supplementary Table 6).

For some lncRNA knockdown experiments, changes in migration ability were consistent with the deregulation patterns of HCC patients. For example, *RP11-166D19.1* was recurrently downregulated in PVTT samples from four patients. The loss-of-function assay showed that knockdown of *RP11-166D19.1* enhanced the migration ability of HCC cells. However, some other lncRNAs, such as *HAND2-AS1*, demonstrated an inconsistent trend between deregulation patterns in HCC patients and experimentally validated functions in cancer cell lines; silencing of *HAND2-AS1* suppressed cell migration, although it was downregulated in 8 of 20 patients' PVTT samples. It has been reported that nearly half of HCC cell lines do not resemble primary tumours^43^, so the intrinsic differences between cancer cells lines and clinical samples might explain the discrepancies between the samples' gene expression patterns and experimentally validated functions in cell lines. Overall, the high validation rate of the candidate lncRNAs showed that the co-expression network, based on previous knowledge of signalling pathways and supplemented by recurrent aberrant expression patterns in matched clinical samples, identified candidate lncRNAs that potentially play functional roles in the sophisticated regulation of cancer development and progression.

## Discussion

Based on an analysis of genomic, epigenomic and transcriptomic data of HCC primary tumours and PVTTs, this study reports several findings. First, based on high-throughput sequencing technology and bioinformatics analysis of 60 matched samples, including primary tumours, PVTTs and adjacent normal tissue, we discovered and characterized an expanded landscape of lncRNAs. Using PVTTs from Chinese HCC patients and deep sequencing data enabled us to detect many candidate lncRNA transcripts. Moreover, we identified lncRNAs that were recurrently deregulated during HCC tumorigenesis and metastasis. Second, integrative multi-omics analysis revealed that recurrent deregulation of lncRNA expression was often associated with alterations in DNA methylation and CNV. In addition, lncRNA expression levels were correlated with clinical data from the TCGA and other published liver cancer data sets. Lastly, using network analysis and loss-of-function assays, we identified functional lncRNAs potentially related to cell adhesion, immune responses and metabolic processes. For example, our paired RNA-seq data showed that lncRNA *HAND2-AS1* was recurrently deregulated; its expression levels among 60 samples were inversely correlated with matching DNA methylation data. Based on our co-expression network, we inferred that *HAND2-AS1* might be related to HCC metastasis. Finally, using an RNAi functional assay, we demonstrated that the function of lncRNA *HAND2-AS1* in HCC cells is related to cell migration.

In addition, we have shown that *RP11-166D19.1* could potentially serve as a promising single-gene HCC biomarker. We also demonstrated that knockdown of *RP11-166D19.1* promoted cell migration. *RP11-166D19.1* is an isoform of lncRNA *MIR100HG*, a leukemia-related oncogene^44^ hosting three miRNAs (*let-7a*, *miR-100* and *miR-125b*) in its introns^45^. As reported previously, lncRNAs are more tissue- and cell-type-specific in comparison with protein-coding genes^18^. Moreover, in comparison with protein-coding genes, the local secondary structures of lncRNAs confer greater stability and provide a greater likelihood of detection^19^. Therefore, translation of these results into candidate lncRNA biomarkers might impact clinical decision-making and ultimately improve clinical outcomes for patients with HCC.

By exploring lncRNA transcriptome alteration, we found that the lncRNA landscapes of PVTTs were indistinguishable from those of matched primary tumours, consistent with previous studies^46^. We employed principal component analysis to assess the expression profiles of recurrently deregulated lncRNAs in different samples. principal component analysis showed that the recurrently deregulated lncRNAs could be used to clearly distinguish primary tumours from adjacent normal tissues, while PVTTs were more similar to primary tumours (Supplementary Fig. 8). This observation showed that the lncRNA expression profile of the PVTTs was very similar to that of their matched primary tumours, consistent with studies on protein-coding genes, CNV and DNA methylation^46^. These findings suggest that (1) primary tumours of HCC patients with PVTT may contain sub-clones with the potential to invade the portal vein and develop into PVTTs; (2) many metastasis-associated lncRNAs were deregulated in these sub-clones. These findings are consistent with clinical observations, because all of our sequenced patients had stage IV HCC with serious PVTT. Although the overall lncRNA expression patterns of PVTTs were similar to those of their matched primary tumours, approximately 100 lncRNAs were significantly and recurrently deregulated in PVTTs in comparison with their expression levels in paired primary tumours. These lncRNAs could play essential roles in metastasis, because they were deregulated further as primary HCC cells invaded the portal vein.

PVTT has been considered as a type of intrahepatic HCC metastasis by several previous genomic studies^10^,^47^. Moreover, PVTTs have also been used to study HCC metastasis in functional and mechanistic studies^48^,^49^, although some researchers have not regarded PVTT as a solid metastatic model. In this genomic study, we provide putative associations and predicted candidates at the transcriptome level, but their functions and the mechanisms in which they play a role must be confirmed by experimental validation.

In this study, we identified recurrently deregulated tumorigenesis- and metastasis-associated lncRNAs, many of which were experimentally validated and mechanistically linked to cancer development and progression. We anticipate that the recurrently deregulated lncRNAs identified in this report could provide a valuable resource for studies aimed at delineating the relationship between functional lncRNAs and HCC tumorigenesis/metastasis. In addition, recent studies suggest that lncRNAs can code for small peptides^50^,^51^. Although COME can detect some of these small peptides^23^, Ribo-seq experiments^52^ are a more reliable way to detect peptides translated from lncRNAs in cancer cells.

## Methods

### Transcriptome assembly for 60 samples from HCC patients

Total RNA from 60 samples from 20 Chinese HCC patients was sequenced (GSE77509). Each patient had three matched samples: primary HCC tumour, adjacent normal liver tissue and PVTT. The patients were ordered using alphabetic labels (A to T) in this paper, but the patients were originally numbered as 3,6, 7, 8, 10, 11, 12, 13, 14, 15, 16, 17, 18, 19, 20, 21, 22, 24, 25 and 26. The PVTT sample of one patient (14) was not distinguishable from normal tissue, so we did not use it in our migration and metastasis analyses. The ethical committee of EHBH hospital approved this study. Informed consent was obtained from each patient.

We first evaluated RNA-seq quality using FastQC (version 0.10.1) and found that all raw reads qualified for the analysis. We aligned the RNA-seq reads to human reference rRNA using Bowtie with one mismatch in order to estimate the rRNA ratio. Most of the rRNAs were removed by our experiments; only a few remained and generated rRNA reads.

Next, the RNA-seq reads were mapped to the human reference genome (hg19) using Tophat (version 2.0.10) (ref. 53) with default parameters. The human genome sequence was downloaded from Ensembl (*Homo sapiens* GRCh37/hg19). After mapping, we further removed PCR duplicates using *rmdup* in Samtools^54^. Further details of the preprocessing results are described in Supplementary Data 1.

Subsequently, based on the mapped reads, we re-assembled a transcriptome using Cufflinks (version 2.2.1) (ref. 55) by providing reference annotations (option ‘-g') from GENCODE (v19) for each data set of 60 samples. Next, we used Cuffmerge^55^ to merge all 60 meta-assemblies to generate a final transcriptome (Supplementary Data 2).

### Identification of candidate lncRNAs

After the transcriptome was assembled, we used several stringent filters to identify a set of candidate lncRNAs, newly assembled lncRNAs, in addition to GENCODE lncRNAs:

1. Transcripts that overlapped (>=1 nt) on the same strand with the exons of protein-coding genes or noncoding RNAs (both canonical ncRNAs and long lncRNAs (lncRNAs)) annotated by GENCODE (V19) were removed. Canonical ncRNAs include rRNA, tRNA, miRNA, snRNA, snoRNA, misc_RNA, mitochondria tRNA and rRNA. Six biotypes were defined as ‘long non-coding RNAs' by GENCODE: ‘lincRNA', ‘processed_transcript', ‘sense_intronic', ‘sense_overlapping', ‘antisense', and ‘3prime_overlapping_ncrna'^13^. Note that ‘processed transcript' means that a transcript does not contain an open reading frame, although it could have a historical protein-coding-style name. Note that some lncRNAs were updated as protein-coding genes in recently released GENCODE annotation versions.
2. Transcripts shorter than 200 bp and without strand information were discarded.
3. To remove fragments of annotated RNA, single-exon transcripts proximal (within 2000, bp) to protein-coding genes or other noncoding RNAs on the same strand were filtered.
4. We calculated transcript expression levels using Cufflinks^55^ with rRNA reads masked. Single-exon transcripts with low expression levels (FPKM <0.5) in all samples were removed.
5. To ensure stringent evaluation of coding potential, we calculated the coding potential of each transcript using two computational tools, CPC (ref. 22) and COME (ref. 23). CPC calculates coding potential based on sequence features, whereas COME integrates expression, RNA secondary structure, conservation and epigenetic signals^56^ (Supplementary Table 1). COME has been successfully applied to noncoding RNA prediction in worm^57^,^58^, fly, human^59^, mouse and *Arabidopsis* 60. Transcripts with CPC score>0 or COME score>0.5 were removed.

### Annotation of candidate lncRNAs

We annotated the genomic locations of the identified candidate lncRNAs, GENCODE lncRNAs and MiTranscriptome lncRNAs (TCGA) by overlapping them with annotated coding genes. Intronic lncRNAs were defined as those located in the intronic regions of coding genes on the sense strand. Antisense lncRNAs were those that overlapped at least 1 nt with any exon (including both coding genes and ncRNAs) on the antisense strand. Cis-lncRNAs (also called sense lncRNAs) were those that were close to (within 2,000 nt of the 5′- or 3′-ends) a protein-coding gene. The remaining lncRNAs that did not overlap with any coding genes or annotated ncRNAs were designated as intergenic lncRNAs.

We also assessed whether any of the candidate lncRNAs overlapped with pseudogenes or transposable elements, because previous studies suggested that some lncRNAs could be derived from such sequences. Annotations of pseudogenes and transposable elements were derived from GENCODE and the UCSC Genome Browser, respectively.

Furthermore, we also annotated lncRNAs with domains/motifs conserved with annotated canonical ncRNAs at the sequence and structure levels. Sequence conservation was assessed by performing BLASTN over canonical ncRNAs sequences. The cutoff E-value was the default value of 1e-5. Secondary structure conservation was calculated by scanning the Rfam structure families of known ncRNAs using INFERNAL/cmscan (*E*-value cutoff was 0.01), in which hits were considered to be sufficiently reliable to be reported in a possible subsequent search round.

### Conservation and SNP enrichment analysis for lncRNAs

The PhastCons scores for multiple alignments of 46 vertebrate genomes were downloaded from the UCSC Genome Browser (https://genome.ucsc.edu/). Two conservation scores were calculated for each transcript; one was based on the average value of the PhastCons scores in the exonic regions, whereas the other was based on those in the intronic regions.

To assess SNPs in different genomic elements, we downloaded two SNP databases: (1) 12,891 SNPs from the National Human Genome Research Institute's GWAS catalogue (https://www.genome.gov/26525384); (2) 14,416,369 common SNPs from dbSNP Build 142 common (downloaded from the UCSC Genome Browser) (treated as background variation). We calculated the number of SNPs that overlapped with the transcripts using the BEDTools *intersect* function. We first calculated the fraction, *frac.(transcripts)*, of the amount of overlapped SNPs from the GWAS catalogue to the number of overlapped background SNPs for different categories of genomic elements (for example, lncRNAs and protein-coding genes). Next, we shuffled the transcripts' positions on the whole genome 100 times and re-calculated *frac.(shuffled transcripts)*. Subsequently, we calculated the odds ratio (OR) as


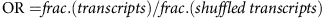


An OR (control) was calculated by replacing SNPs from the GWAS catalogue with control SNPs:


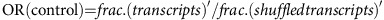


where the control SNPs were randomly selected from the background SNPs shuffled over the whole genome. The significance of comparison for the OR over OR (control) was tested via paired Student's *t*-test.

### Differential expression analyses

From 8,603 newly assembled lncRNAs and 13,870 known lncRNAs annotated in GENCODE, we identified lncRNAs that were differentially expressed between primary tumours and adjacent normal tissues, as well as between PVTTs and matched primary tumours. We used two different strategies: treating all patients as biological replicates (DESeq2 (ref. 29) and Wilcoxon signed-rank test) and treating each patient individually (GFOLD (ref. 28)) followed by recurrence count.

In DESeq2 and Wilcoxon signed-rank test, significantly differentially expressed lncRNAs were defined as satisfying two criteria: |log2 (fold-change)|>1 and *q*-value (Benjamini-Hochberg adjusted *P* value)<0.05.

In GFOLD (V1.1.3), which was especially useful for assessing samples without replicates, differentially expressed lncRNAs were first identified for each individual patient. GFOLD calculated its own statistics (that is, significance cutoff and GFOLD value) for expression level changes based on the posterior distribution of the log fold-changes in expression^28^. The significance cutoff was set at 0.01 (-sc 0.01), also requiring |GFOLD value|>1.

After differentially expressed lncRNAs were identified in individual patients using GFOLD (Supplementary Fig. 6), lncRNAs that were not recurrently deregulated in multiple patients were filtered out from the results, yielding 1,112 lncRNAs that were recurrently differentially expressed in at least 8 of 20 patients (permutation test, FDR<0.05) when primary tumours were compared with adjacent normal tissues, as well as 107 lncRNAs that were recurrently differentially expressed in at least four patients (permutation test, FDR<0.05) when PVTTs were compared with matched primary tumours (Supplementary Fig. 7). We used a permutation test to estimate the FDR of the recurrence among multiple patients. First, we randomly sampled size-matched lncRNAs for each patient according to the number of differentially expressed lncRNAs identified by GFOLD and calculated the number of recurrences. Next, we repeated the sampling and counting 10,000 times to estimate a null distribution for the number of recurrences. Finally, we calculated the FDRs for the recurrences based on the null distribution. We set the minimum number of recurrences to 8 and 4 for primary tumours versus adjacent normal tissues and PVTTs versus primary tumours, respectively, to ensure that the FDR of each recurrence was smaller than 0.05.

### Integrative analyses of TCGA LIHC data

We downloaded RNA-seq data for 157 LIHC patients with clinical data in the TCGA from the NCI Cancer Genomics Hub (CGHub)^61^. We calculated the expression level of each gene/lncRNA (FPKM) for each TCGA sample using Cufflinks^55^.

In the Kaplan-Meier survival analysis, the survival data included vital status, days to death, and other variables, which were available for 151 of 157 patients. We first divided the samples into two groups (51 low-expression and 100 high-expression) according to the expression level of a marker gene/lncRNA (for example, *RP11-166D19.1*). Next, we used Kaplan-Meier survival analysis^62^ to perform a 5-year survival analysis via the *survival* package (https://cran.r-project.org/web/packages/survival) in the R environment for statistical computing and computed significance using the log-rank test.

Additional clinical information (age, gender, AFP, serum albumin, prothrombin time, cirrhosis, vascular invasion, and so on) for the LIHC patients in the TCGA was downloaded for the multivariate analysis. Based on two groups defined by the expression level of a particular lncRNA (for example, *RP11-166D19.1*), the odds ratio of each clinical criterion was calculated for each class of patients (low expression and high expression). A forest plot was drawn with odds ratios and 95% confidence intervals for each clinical criterion.

We used GSEA (v2.0.13) (ref. 63) to assess enrichment of sets of recurrently deregulated lncRNAs in other data sets. GSEA requires three input files: a gene set, expression data and phenotype labels. We used the recurrently deregulated lncRNA set (the tumorigenesis set or metastasis set) as the gene set. Expression data were derived from the TCGA cohort or published liver cancer data^21^. For the published data, we used sample information (adjacent normal tissue, primary tumour and PVTT) as phenotype labels. Because the TCGA LIHC cohort had no PVTT or other metastasis samples, we classified primary tumour samples into invasion and non-invasion groups based on clinical information (T stages in the TNM staging system: T1 versus T2–T4). The lncRNAs were rank-ordered by differential expression (signal2Noise in GSEA v2.0.13) (ref. 63) between the two groups.

### Subtypes of HCC tumours

Based on the expression pattern of 619 signature genes, HCC primary tumours were classified into three subclasses, S1, S2 and S3 (refs 31, 32). We used an online classification tool, NearestTemplatePrediction, from GenePattern (http://software.broadinstitute.org/cancer/software/genepattern/modules) to perform the classification. Finally, the Wilcoxon rank-sum test was used to identify lncRNAs deregulated in each subclass (Supplementary Fig. 10) (q-value<0.05 and fold change>2).

### Copy number variation data for lncRNAs

DNA copy numbers were determined for the 60 matched samples (PVTT/tumour/normal tissue samples from 20 patients) using Affymetrix CytoscanHD arrays by following the manufacturer's protocol (GSE77275). The CytoscanHD array contains 2,696,550 probes, including 1,953,246 nonpolymorphic probes. The GISTIC algorithm (GISTC2.0) (ref. 33) was used to calculate G-scores and FDRs (q-values) for the aberrant regions and thus identify genomic regions that were significantly amplified or deleted across all samples. G-scores consider the amplitude of the aberration and its frequency of occurrence across all samples. Aberrant regions were considered significant when the assigned FDR q-value was less than 0.25. The GISTIC algorithm also reported genes found in each aberrant region. We identified recurrently deregulated lncRNAs for which CNVs contributed to their deregulation.

### DNA methylation data for lncRNAs

DNA methylation profiles were probed using the Illumina Infinium HumanMethylation 450 k BeadChip, which contains more than 485,000 CpG sites (GSE77269). *β* values were calculated to independently assess the methylation levels of the CpG sites for each data set. CpG sites were distributed across the promoters and gene bodies of the lncRNAs. To identify recurrently deregulated lncRNAs that were epigenetically regulated by DNA methylation, we assigned all CpG sites corresponding to promoter regions (2,000 bp upstream of the TSS) to each lncRNA. Pearson correlation coefficients between expression levels and *β*-values were calculated for each lncRNA and all assigned methylation sites across all 60 samples. When there were multiple CpG sites for the same gene promoter, the CpG site with the highest correlation was assigned to that lncRNA. Recurrently deregulated lncRNAs with Pearson correlation coefficients <−0.3 were identified as lncRNAs regulated by DNA methylation alteration.

### Co-expression network construction

We adapted a published method^35^ to construct a co-expression network of lncRNAs (including both GENCODE lncRNAs and lncRNAs identified in the HCC samples). The expression levels derived from the total RNA-seq data for the 60 samples were used. Genes with a maximum expression level among all data sets that ranked in the bottom 20% were excluded from the input gene list. For each gene pair (including lncRNAs and protein-coding genes), we calculated the Pearson correlation coefficient and corresponding *P* -value using the *WGCNA* package for the R Environment for Statistical Computing^64^. All *P* values were adjusted for multiple testing via Bonferroni correction in the *multtest* R package^65^. Markov clustering (MCL)^66^ was used to detect highly inter-connected gene/lncRNA clusters. Bonferroni-adjusted *P* values (cutoff: 0.01) were used as edge weights for MCL. To control the size of the clusters generated from the MCL clustering, the inflation coefficient was set to 2.4.

### Gene ontology and pathway enrichment analyses

For the protein-coding genes in each co-expression cluster, we used R package *topGO* (ref. 67) to estimate enrichment in biological process (BP) terms for different gene sets. We estimated the significance of GO term enrichment using a hypergeometric test. Moreover, we used R package *KEGGREST* (ref. 68) to estimate enrichment in biological pathways for each cluster. We also annotated 271 driver genes of liver cancer, which were derived from a recent study^39^.

### Knockdown of candidate lncRNAs and transwell migration assay

Three human liver cancer cell lines, HepG2, SMMC-7721 and HCCLM9, were used to conduct functional assays (HepG2 was purchased from the American Type Culture Collection (ATCC, Manassas, VA, USA), SMMC-7721 and HCCLM9 were provided by Professor Jianzhong Xi from Peking University). All cell lines were tested for mycoplasma contamination and no contamination was found. Three siRNAs were designed for each candidate lncRNA (Supplementary Table 3) and obtained from GenePharma. qRT-PCR was used to monitor siRNA knockdown efficiency. Primer sequences used for qRT-PCR are listed in Supplementary Tables 3 and 4. Transwell migration assays were used to test the effects of candidate lncRNAs on cell migration. CCK8 cell proliferation assays were used to assess the effects of candidate lncRNAs on cell proliferation.

For the cell migration assays, cells were first transfected with 30 nM siRNA mixtures of three designed siRNAs in 24-well plates and incubated for 48 h, followed by resuspension and washing with phosphate-buffered saline buffer. Next, for each experiment, approximately 40,000 cells were seeded into the upper chamber of a transwell insert (pore size, 8 μm, Costar) in 100 μl of serum-free medium per well. Medium (600 μl) containing 10% serum was placed in the lower chamber to act as a chemoattractant. The seeded cells were incubated for 24 h to allow them to migrate. Subsequently, non-migratory cells were removed from the upper chamber by scraping it with cotton. The cells remaining on the lower surface of the insert were fixed with 4% formaldehyde (Sigma) and stained with DAPI for counting. Each type of cell was assayed in triplicate. Moreover, we repeated the RNAi knockdown and migration assays for all candidate lncRNAs using each siRNA at the same concentration.

To assess whether cell proliferation affected cell migration activity in the transwell assays, we performed CCK8 cell proliferation assays after knockdown of candidate lncRNAs using each individual siRNA. For the cell proliferation assays, cells were transfected with 30 nM of each siRNA and allowed to grow for 48 h. Next, cells were incubated with 10% CCK8 reagent (DoJinDo Laboratories, Japan) for 1 h at 37 °C. The absorbance of the solution in each well at 450 nm was detected using an automatic spectrometer (Multimode Reader; Enspire). Each experiment was performed in triplicate.

### Data availability

The high-throughput sequencing data from this study have been submitted to the NCBI Gene Expression Omnibus (GEO, http://www.ncbi.nlm.nih.gov/geo/) under accession number GSE77276 (RNA-seq, GSE77509; small RNA-seq, GSE76903; 450k array, GSE77269; Cytoscan HD array, GSE77275). The authors declare that all other data are available in the article and its Supplementary Information Files or from the corresponding author on reasonable request.

## Additional information

**How to cite this article:** Yang, Y. *et al*. Recurrently deregulated lncRNAs in hepatocellular carcinoma. *Nat. Commun.* **8,** 14421 doi: 10.1038/ncomms14421 (2017).

**Publisher's note:** Springer Nature remains neutral with regard to jurisdictional claims in published maps and institutional affiliations.

## Supplementary Material

Supplementary Information Supplementary Figures, Supplementary Tables and Supplementary References

Supplementary Data 1 RNA-seq data preprocessing and mapping results

Supplementary Data 2 Coordinates of newly assembled lncRNAs

Supplementary Data 3 Newly assembled lncRNAs' genomic positions and overlapped genomic elements

Supplementary Data 4 Expression of newly assembled lncRNAs in 60 samples The FPKM value of each lncRNA was calculated by Cufflinks at gene level

Supplementary Data 5 Expression of GENCODE lncRNAs in 60 samples The FPKM value of each lncRNA was calculated by Cufflinks at gene level

Supplementary Data 6 Recurrently deregulated lncRNAs in 60 samples (Recurrently deregulated, DESeq2, Wilcoxon, GFOLD+Recurrence)

Supplementary Data 7 Significantly deregulated lncRNAs in each HCC subclass

Supplementary Data 8 Recurrently deregulated lnRNAs driven by CNV and/or DNA methylation

Supplementary Data 9 Gene and lncRNA lists and clusters in co-expression network
