# Supplementary material for: Recurrently deregulated lncRNAs in hepatocellular carcinoma
Source: Nat Commun. 2017 Feb 13;8:14421. doi: 10.1038/ncomms14421 (PMC5316832; doi:10.1038/ncomms14421)
Supplement: Supplementary Information — Supplementary Figures, Supplementary Tables and Supplementary References [file ncomms14421-s1.pdf]

Supplementary Information

A. Transcript level

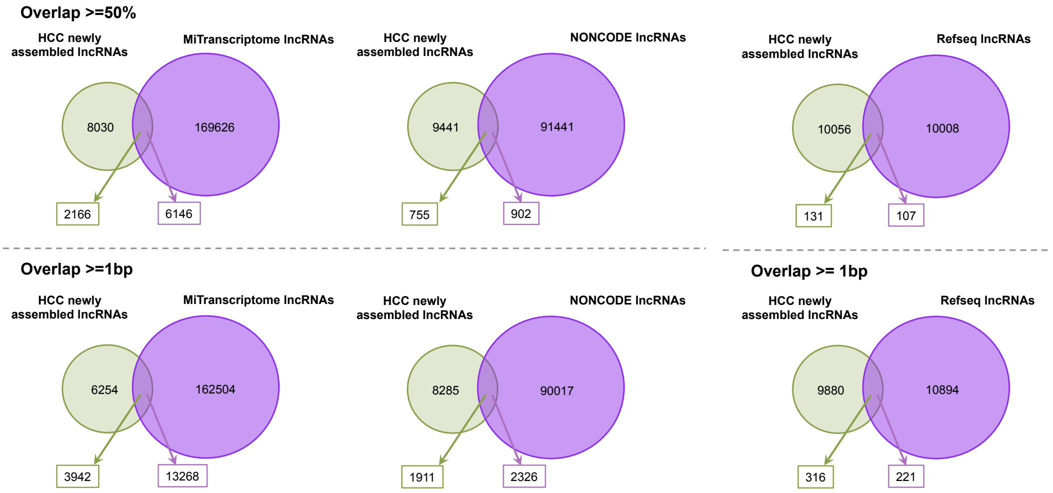

B. Gene level

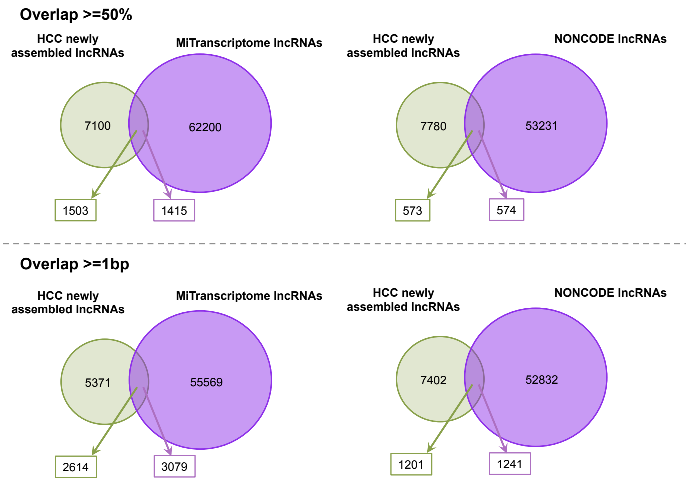

Supplementary Figure 1. Overlap of newly assembled lncRNAs with other lncRNA sets: MiTranscriptome (TCGA), NONCODE and RefSeq lncRNAs. The MiTranscriptome lncRNAs were downloaded from a study on TCGA poly(A)+ RNA-seq data[1]. The NONCODE lncRNAs were downloaded from NONCODE database (V4)[2]. The Refseq lncRNAs were downloaded from NCBI (Release 72) (http://www.ncbi.nlm.nih.gov/refseq)[3]. The numbers in **(A)** are counting on transcripts, the numbers in **(B)** are counting on genes, each of which could include multiple transcripts, because Refseq only provides lncRNA annotation in transcript format. The numbers in the upper panel of each plot are counted when the overlapping is larger or equal to 50% of the gene/transcript length. The numbers in the lower panel are counted when overlapping is larger or equal to 1bp.

A.

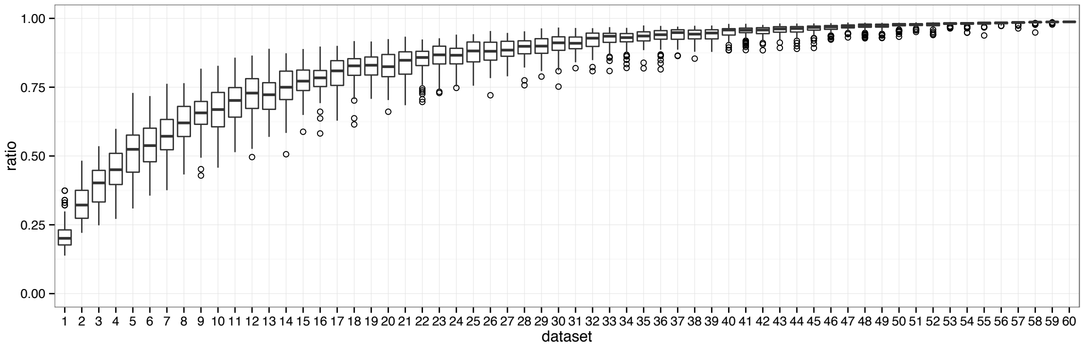

B.

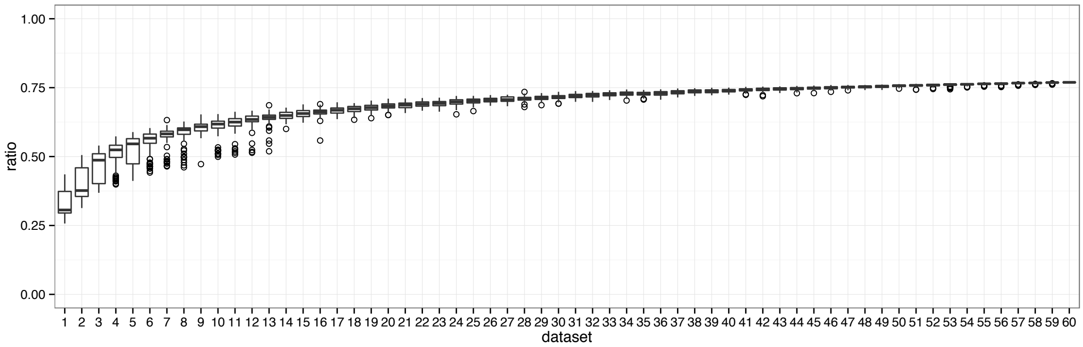

C.

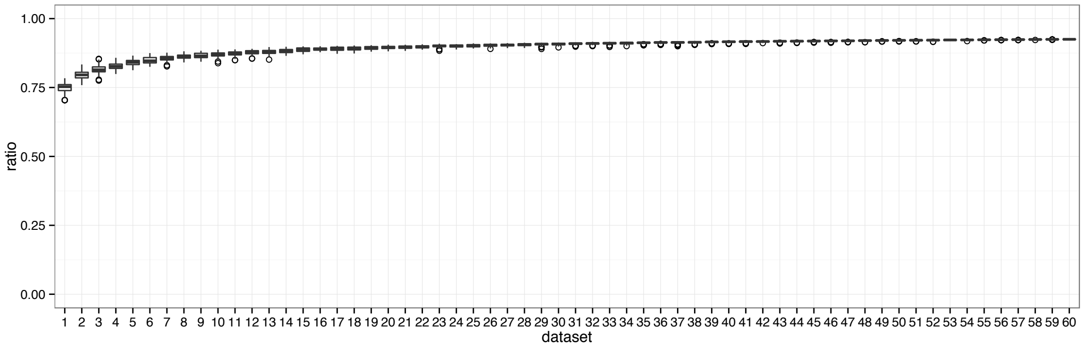

Supplementary Figure 2. Saturation plots in the 60 samples we sequenced. 60 datasets were used to calculate the expression ratios of newly assembled lncRNAs, GENCODE lncRNAs and protein-coding genes, The FPKM >=0.1 is set as the expression cutoff. Each point at a given number of considered datasets corresponds to different combination of datasets. The figure depicts saturation plot for newly assembled lncRNAs (**A**), GENCODE lncRNAs (**B**) and protein-coding genes (**C**)

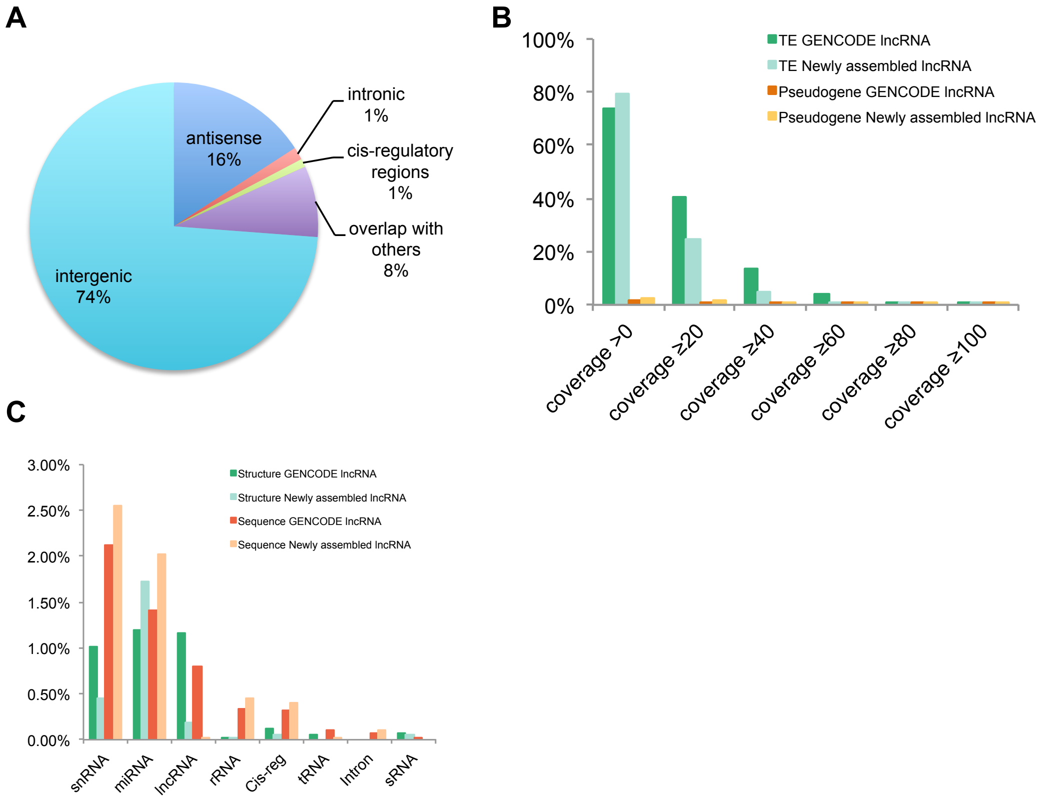

Supplementary Figure 3. LncRNAs' genomic positions and overlapped genomic elements. (**A**) Classification of newly assembled lncRNAs according to their genomic locations with respect to GENCODE v19. Antisense, overlapped with protein-coding genes (including exonic, intronic, up- and down- stream 2K regions) in the antisense strand. Intronic, located within the intronic regions of protein-coding genes in same strand. Cis-regulatory region, overlapped with cis-regulatory regions (up- and down- stream 2K regions, no exonic overlap) of protein-coding genes in the same strand. Overlap with others, overlapped with other types of annotated transcripts in either strand. Intergenic, all remaining lncRNAs. (**B**) Overlap with pseudogenes and transposable elements. The annotation of transposable elements is downloaded using UCSC table browser. The pseudogene annotation is from GENCODE v19. All lncRNAs overlapped these elements in either strand is counted. Coverage is calculated as the length of overlapped exonic regions to the length of lncRNAs. The ratio of all lncRNAs overlapped with TE (coverage>0) is nearly 80%, which is consistent with previous studies[4]. (**C**) Number of lncRNAs containing conserved domains/motifs at sequence and structure levels. Structure search to conserved RNA models in Rfam is performed using *cmscan*. Sequence search to conserved RNA models in Rfam is performed using *blast*.

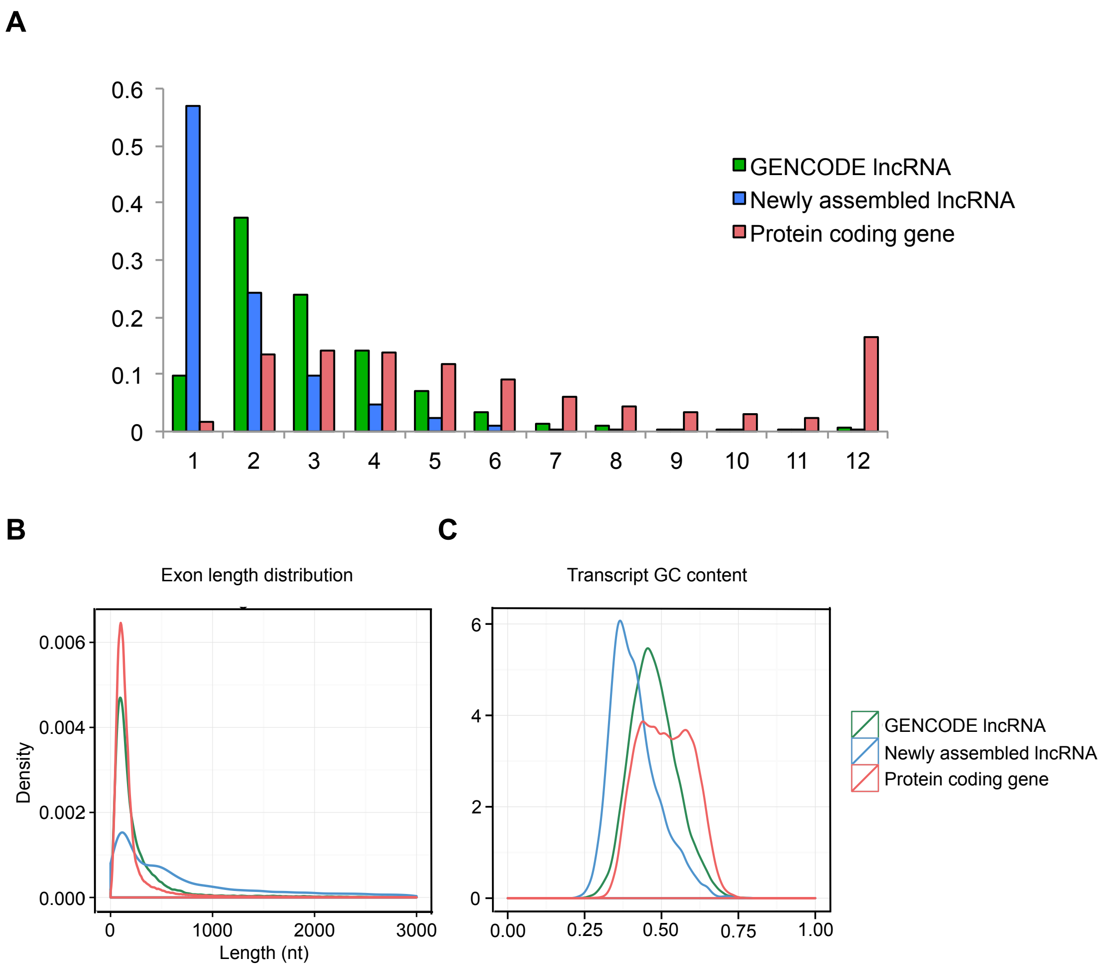

Supplementary Figure 4. Additional characterization of lncRNAs. Additional characteristics comparison among GENCODE lncRNA, newly assembled lncRNA and protein-coding genes. (**A**) The number of exons per transcript. (**B**) Exon size distribution. (**C**) Transcript GC content.

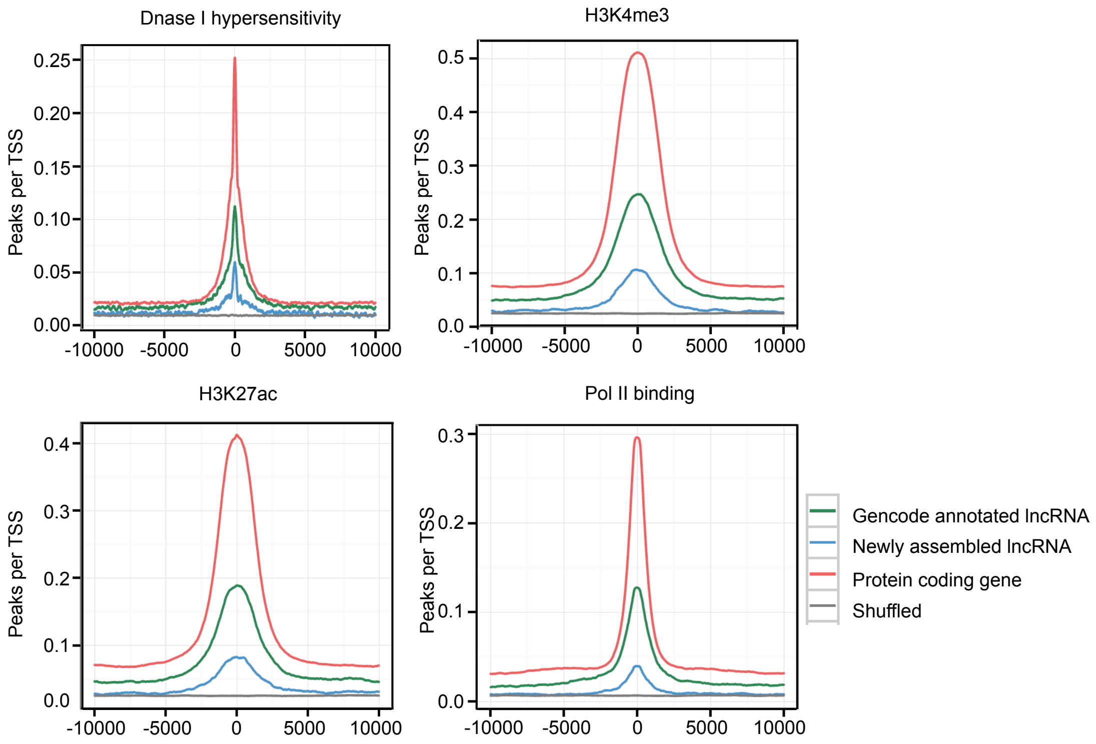

Supplementary Figure 5. Aggregation plots for signals of active markers. We utilized ENCODE Project data sets[5] to show the signal enrichment of different modification signals around the transcription start site (TSS) of different kinds of transcripts. The processed signals of H3K4me3, H3K27ac, DNase I hypersensitivity and Pol II binding data of XX cell lines were downloaded from the UCSC Genome Browser (https://genome.ucsc.edu/). The aggregation plots were draw around each TSS (upstream and downstream 10K nt) for each gene category (e.g. lncRNA, protein-coding gene, etc).

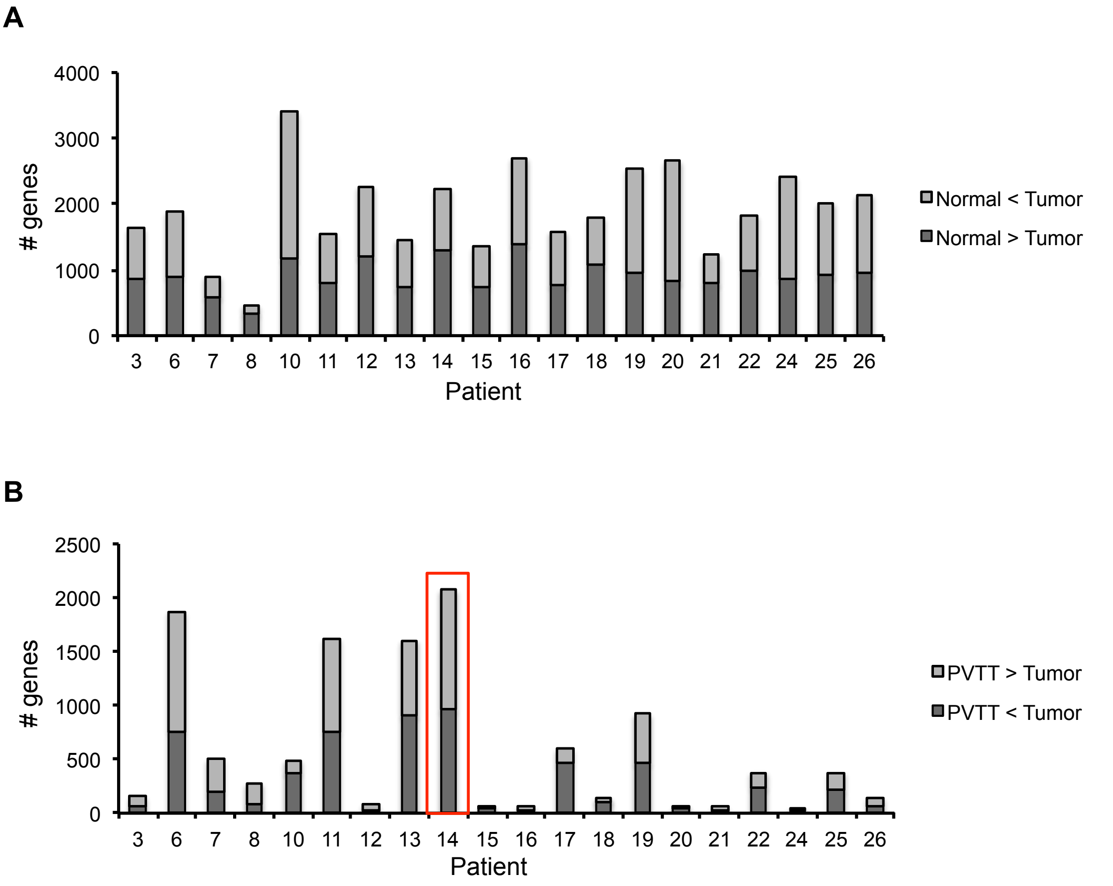

Supplementary Figure 6. Differentially expressed lncRNAs in individual patients defined by GFOLD. The bar plots depict the number of differentially expressed lncRNAs in each individual patient. (**A**) Tumorigenesis-associated, that is, differentially expression between primary tumor and adjacent normal tissue. (**B**) Metastasis-associated, that is, differentially expression between primary tumor and PVTT. Patient 14 is not included for PVTT because the sample was contaminated.

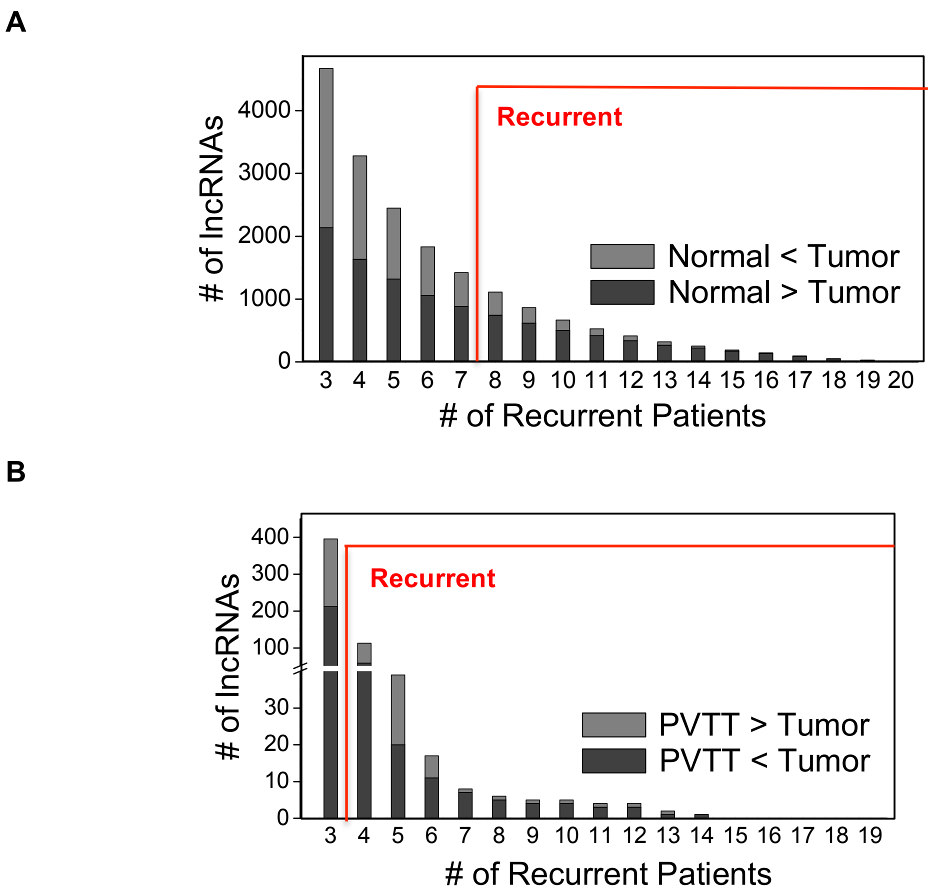

Supplementary Figure 7. Recurrence of the differentially expressed lncRNAs defined by GFOLD. The bar plots depict the recurrence of differentially expressed lncRNAs. (**A**) Tumorigenesis associated, that is, differentially expressed lncRNAs between adjacent normal tissues and primary tumors, including normal > tumor and normal < tumor; (**B**) Metastasis associated, that is, differentially expressed lncRNAs between primary tumors and PVTTs, including tumor > PVTT and tumor < PVTT. The axis is the number of patients in which the lncRNAs were differentially expressed. Patient 14 is not included for PVTT because the sample was contaminated.

A

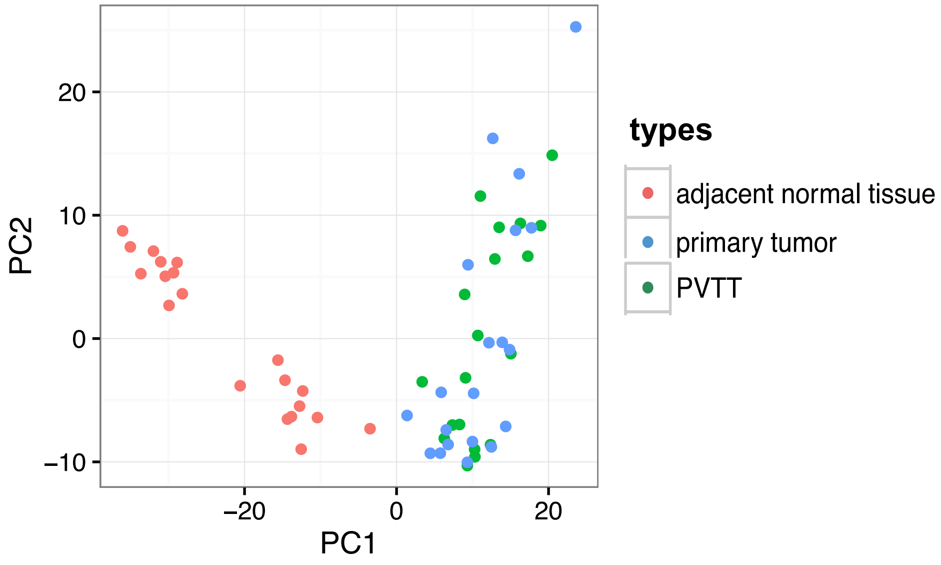

B

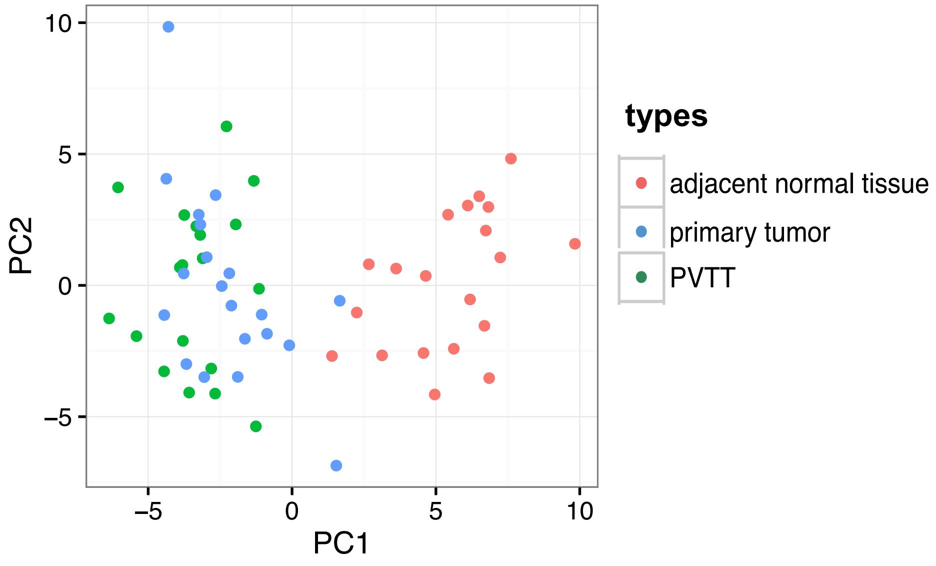

Supplementary Figure 8. PCA based on the expression profile of recurrently deregulated lncRNAs. (**A**) Tumorigenesis-associated recurrently deregulated lncRNAs. (**B**) Metastasis-associated recurrently deregulated lncRNAs.

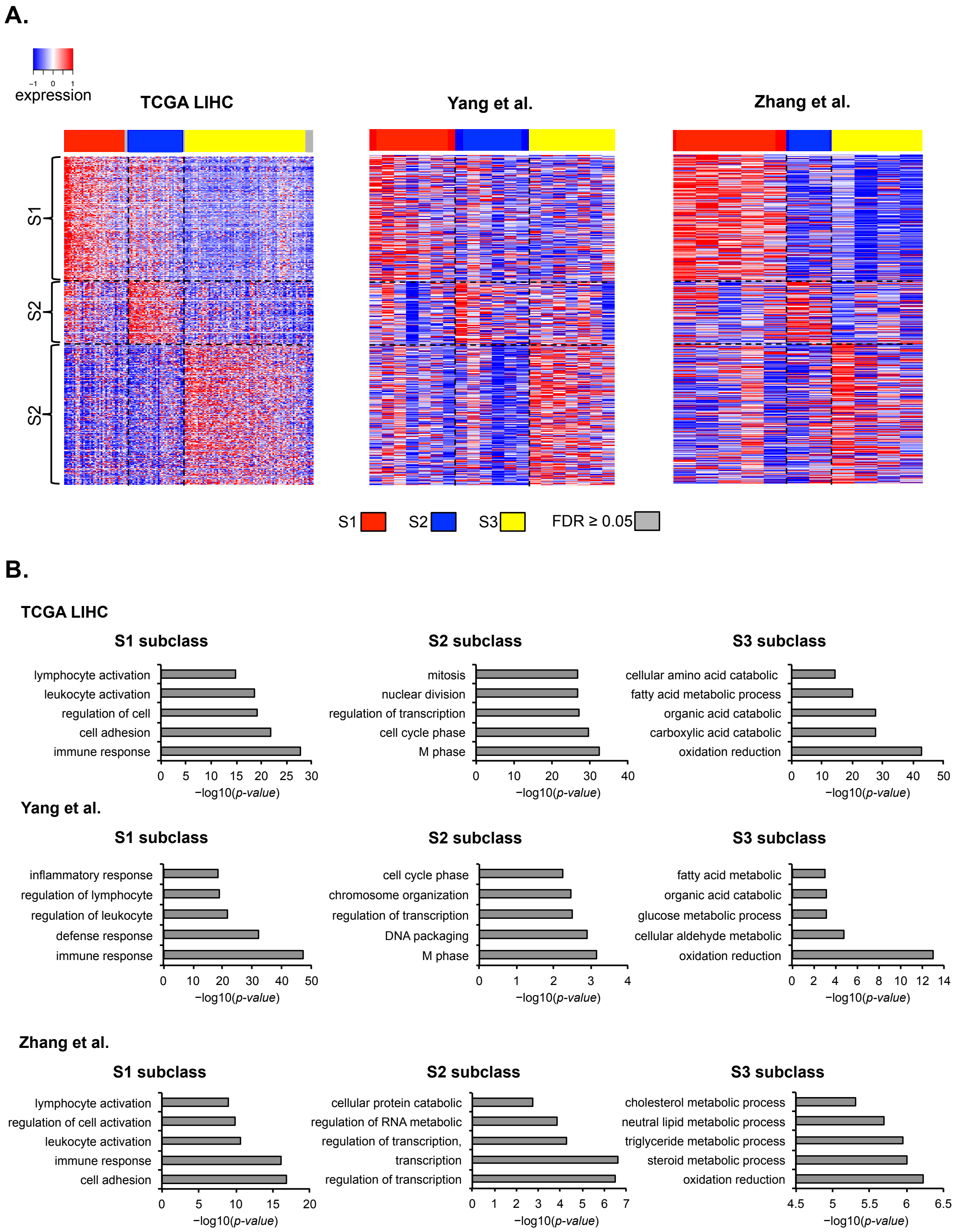

Supplementary Figure 9. Molecular classification of HCC. The HCC tumors from TCGA LIHC study, our study (Yang et al.) and another published study (Zhang et al.)[6] were clustered into three HCC subclasses (S1, S2 and S3) based on 619 signature genes[7, 8]. (**A**) The heatmaps depict the gene expression profile of the 619 genes in each HCC subclass. High and low expression levels are represented by red and blue color in the heatmaps, respectively. Predicted subclasses are shown in the colorbar using red (S1), blue (S2), yellow (S3) and the samples with low confident prediction

(FDR ≥ 0.05) were labeled in grey. (**B**) The bar plots depict GO enrichment for the upregulated protein-coding genes in each HCC subclass, which was consistent with previous study[7, 8].

A B

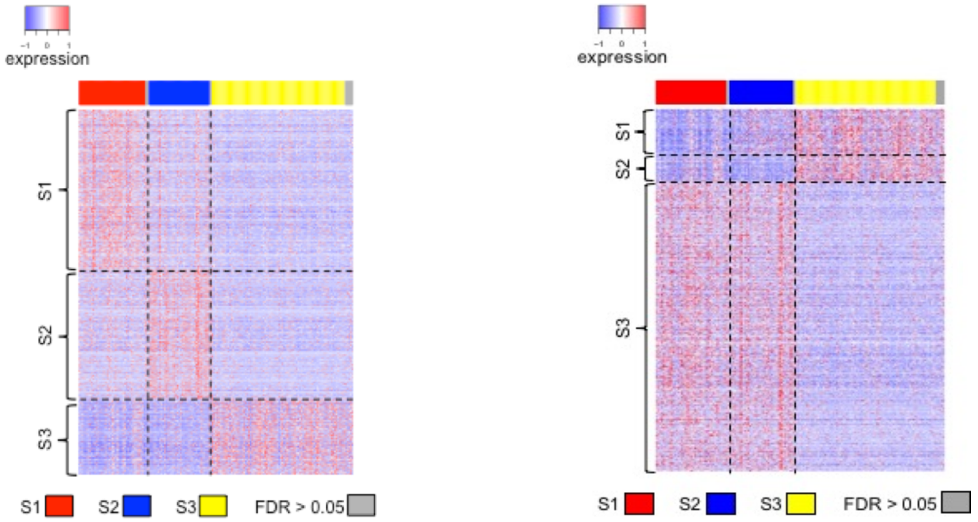

C

	S1		S2		S3	
	upregulated	downregulated	upregulated	downregulated	upregulated	downregulated
Total	581	236	464	114	355	1404
Recurrently upregulated lncRNAs associated with tumorigenesis	16	0	31	1	1	74
Recurrently downregulated lncRNAs associated with tumorigenesis	13	82	1	45	133	15
Recurrently upregulated lncRNAs associated with Metastasis	7	3	6	1	1	16
Recurrently downregulated lncRNAs associated with Metastasis	8	7	2	7	11	12

Supplementary Figure 10. Significantly deregulated lncRNAs in each subclass. Significantly deregulated lncRNAs in each HCC subclass. The upregulated (**A**) and downregulated (**B**) lncRNAs were defined by Wilcoxon rank-sum tests (q-value < 0.05 and fold change > 2) on TCGA expression data, comparing one subclass with the other two. High and low expression levels are represented by red and blue color in the heat-maps, respectively. Predicted subclasses are shown in the color bar using red (S1), blue (S2), yellow (S3) and the samples with low confident prediction (FDR ≥ 0.05) were labeled in grey. (**C**) Summary of total lncRNAs, recurrently deregulated lncRNAs associated with tumorigenesis and recurrently deregulated lncRNAs associated with metastasis, which were deregulated in each subclass. The details were listed in Supplementary File 7.

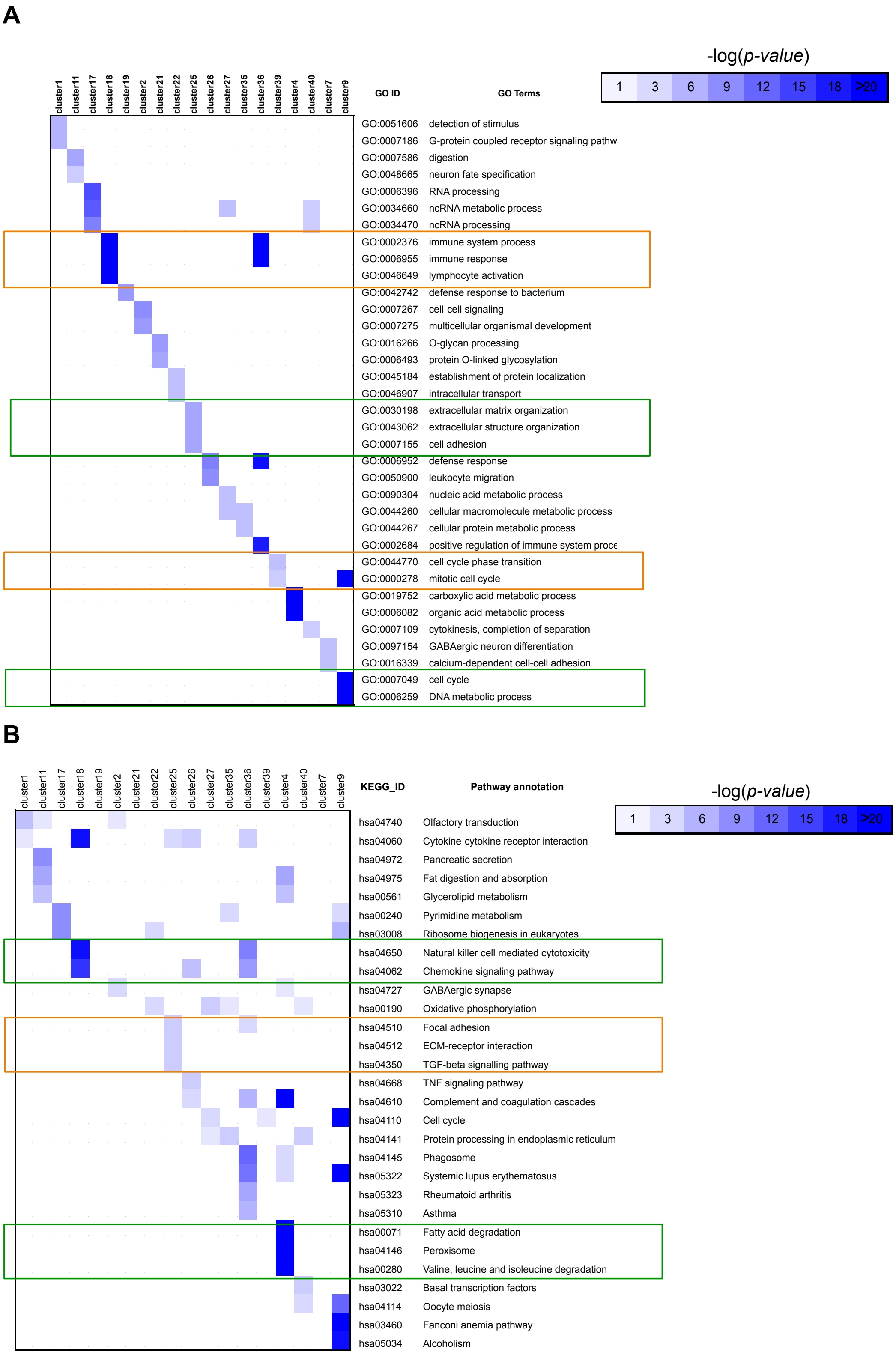

Supplementary Figure 11. GO and Pathway enrichment heatmap for all subclusters defined by co-expression network. Function enrichment for protein-coding genes from various sub-clusters which defined by co-expression network. (**A**) GO enrichment; (**B**) KEGG pathway enrichment.

A

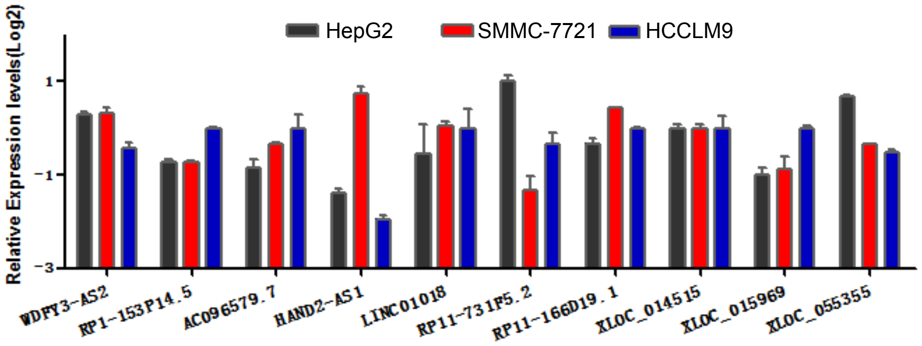

B

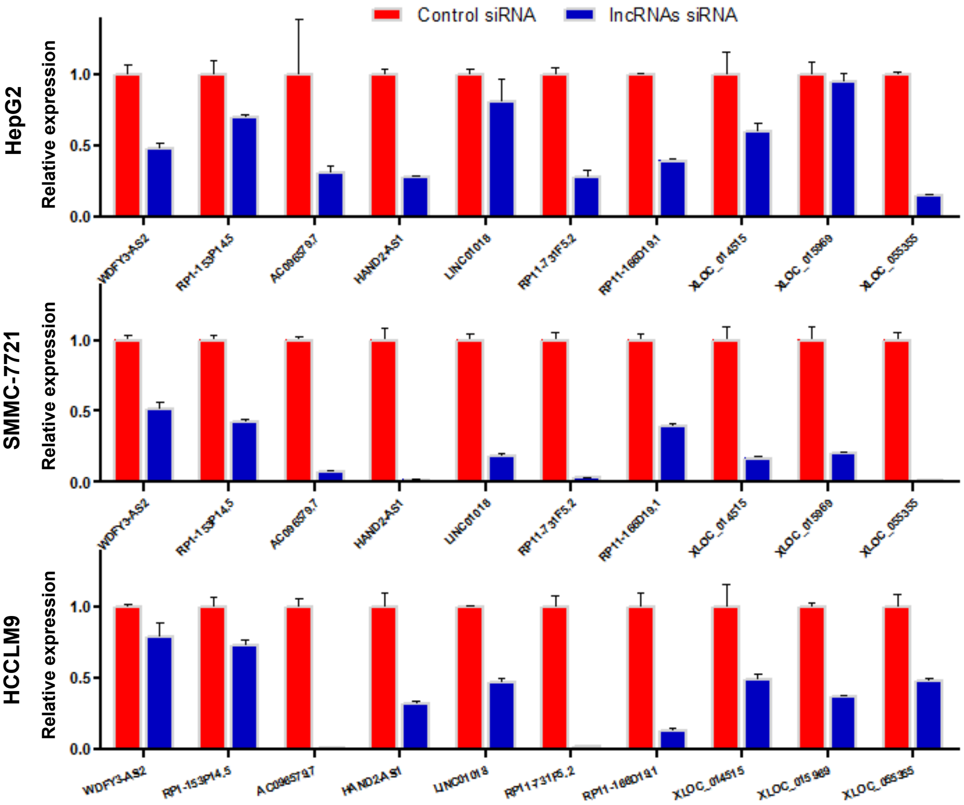

Supplementary Figure 12. Knockdown of candidate lncRNAs in three liver cancer cell lines using siRNA mixture. Knockdown of candidate lncRNAs in three liver cancer cell lines using siRNA mixtures. (**A**) The relative expression levels of all 10 candidate lncRNAs in three liver cancer cell lines, including HepG2 (black), SMMC-7721 (red) and HCCLM9 (blue) cell lines. (**B**) The relative expression levels of all candidate lncRNAs after knockdown using siRNA mixture (blue) or after treatment with control siRNA (red). Error bars represent the s.d. of three experiment replicates.

A

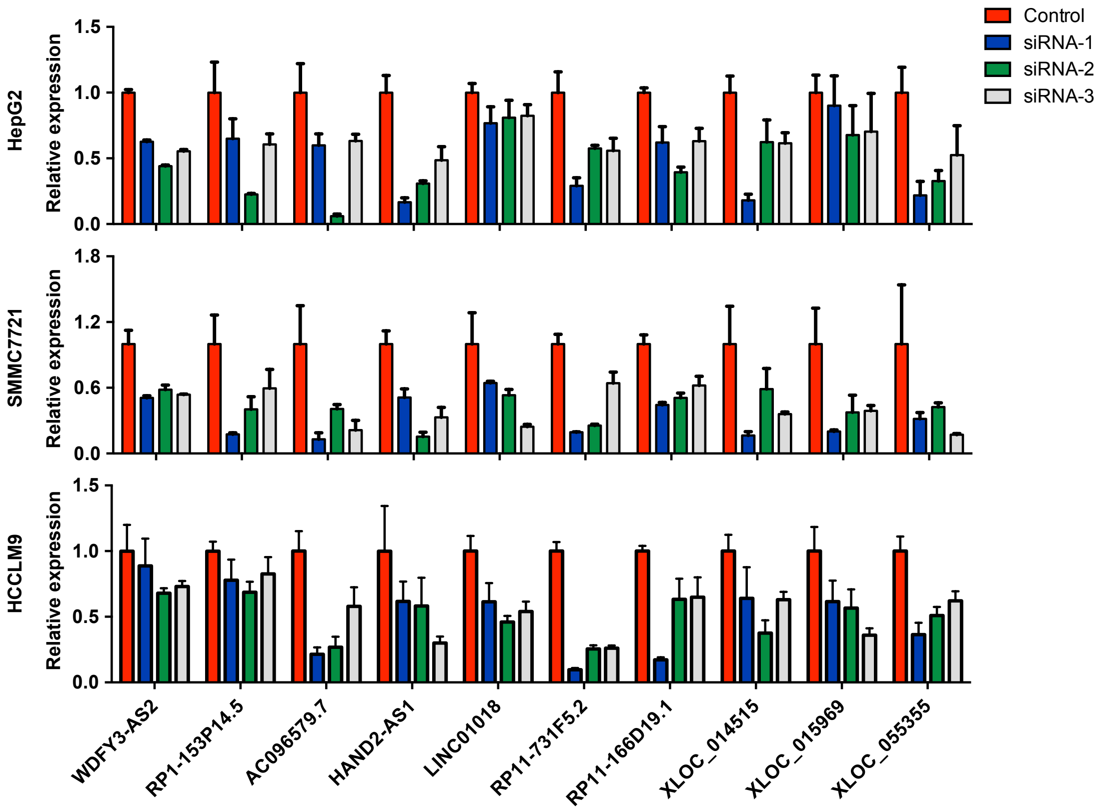

B

lncRNA	HepG2				SMMC-7721				HCCLM9			
	mixture	siRNA-1	siRNA-2	siRNA-3	mixture	siRNA-1	siRNA-2	siRNA-3	mixture	siRNA-1	siRNA-2	siRNA-3
WDFY3-AS2	*	*	**	***	***	**	*	*				
RP1-153P14.5					**	**	*	*				
AC096579.7												
HAND2-AS1					**	**	**	**	**	**	**	**
LINC01018												
RP11-731F5.2												
RP11-166D19.1	*	*	**	*					*	*	*	*
XLOC_014515									**	*	*	*
XLOC_015969									**	*	*	*
XLOC_055355									**	**	*	**
Negative Control												

0.4 0.6 0.8 1 1.2 1.4 1.6

Fold change of migration cell numbers

*** p-value<0.001 **p-value<0.01 *p-value<0.05

C.

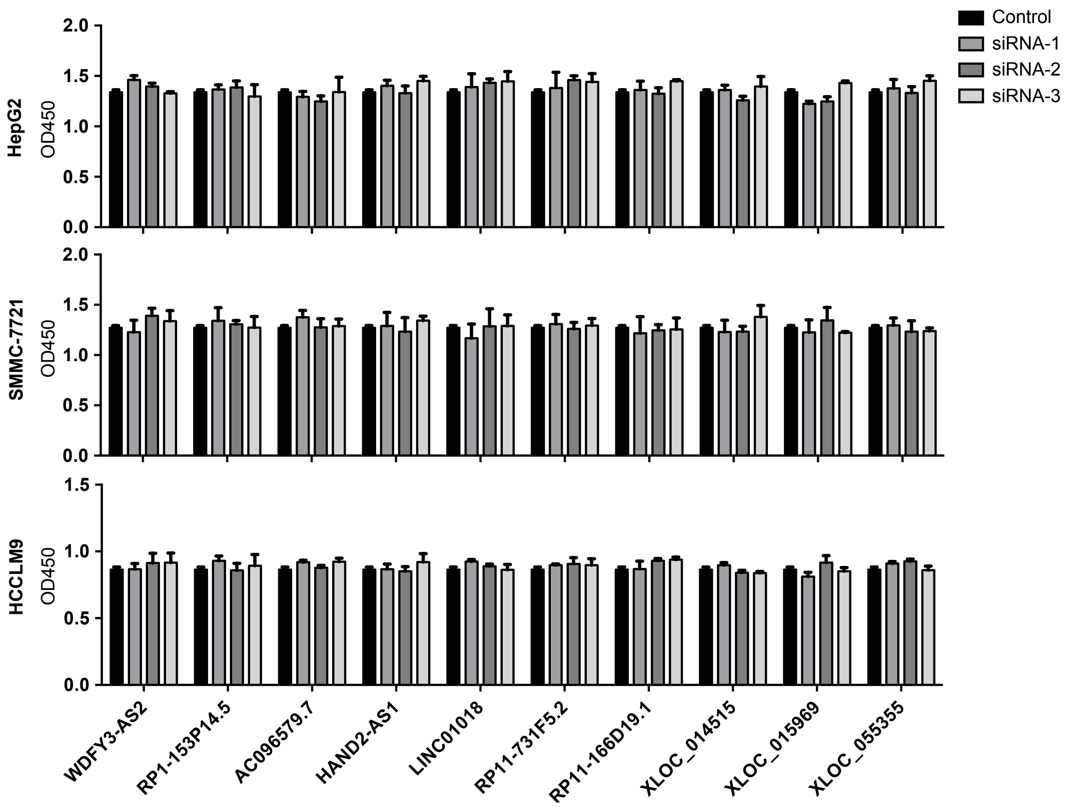

Supplementary Figure 13. Knockdown of candidate lncRNAs in three liver cancer cell lines using three siRNA separately. Knockdown of candidate lncRNAs in three liver cancer cell lines using three siRNA separately. (**A**) The relative expression levels of all candidate lncRNAs after knockdown using all three siRNA separately or after treatment with control siRNA in three liver cancer cell lines (HepG2, SMMC-7721 and HCCLM9). (**B**) The heatmap depicting the transwell migration assays for all candidate lncRNAs. The values in the heatmap showing the fold-change (*P*-value<0.05) of the migration cell numbers of knockdown cells using siRNA mixtures and three siRNAs separately over those of control cells. All results are expressed as the mean derived from three independent experiments. Student's unpaired *t*-test was used for comparison of knockdown cells and control cells. Details of the heatmap values are shown in Supplementary File 10. *P-value<0.05, **P-value<0.01, ***P-value<0.001, *t*-test, n=3. (**C**) The barcharts showing the CCK8 cell proliferation assays for all candidate lncRNAs. The column values represent OD450 values of three liver cancer cell lines after knockdown of candidate lncRNAs using three siRNAs separately or after treatment with control siRNA. We repeated every siRNA's experiment three times; and details of all values are shown in Supplementary File 11. Error bars represent the s.d. of three experiment replicates.

Supplementary Tables

Supplementary Table 1. Filtering steps for identifying newly assembled lncRNAs

Step		Parameters	Transcript	
**Filter**	0.Merged transcriptome	NA	393,479	
	1.Filter by annotation[1]	No overlap	73,067	
	2.Filter by length	length > 200bp	72,065	
	3.Filter by strand	strandness	68,095	
	4.Annotate by exon number	exon number	mono-exonic	multi-exonic
			51,535	16,560
	5.filter by distance	distance > 2000bp	46,726	
	6.filter by expression	FPKM > 0.5 in at least 1 sample	9,908	
	7.novel transcript	NA	26,468	
	8.Filter by CPC	default	10,449	
	9.Filter by COME	default	10,196	
**Total newly assembled lncRNA**			**10,196 (8,603 gene)**	

[1]We used protein-coding genes (exons) and lncRNAs (exons) annotated in Gencode (V19) as the known annotations.

Supplementary Table 2. Candidate lncRNAs for loss-of-function assay

lncRNAs name	ENSEMBL ID	lncRNA significantly correlated cell adhesion genes	cluster
WDFY3-AS2	ENSG00000180769	CD4, SRPX, BMX, ENG, CXCL12, STAB2, MMRN1, DPT, ITGAD ,BMP10, MFAP4, TNXB, DSCAM	25
RP1-153P14.5	ENSG00000227920	LY9, ITGA9, LAMA2	18
**AC096579.7**	ENSG00000231486	CD6, EPHA3 ,LAMC3, SRPX, AEBP1, ENG, CXCL12, SELPLG, CPXM2, LY9, OMD, ISLR, EMILIN1, MMRN1 ,DPT ITGA9, CD226, CD96, SVEP1, MFAP4, TNXB, SELP, LAMA2	18
**HAND2-AS1**	ENSG00000237125	EPHA3, LAMC3, SRPX, BMX, ENG, CXCL12, CPXM2, CLDN10, STAB2, MMRN1, DPT, ITGA9, CD226, BMP10, SVEP1, MFAP4, TNXB, SELP, LAMA2	25
**LINC01018**	ENSG00000250056	CDHR5, MPDZ, GNE, AZGP1, DSCAM, COL18A1	4
**RP11-731F5.2**	ENSG00000253364	CD6, SIGLEC6	25
**RP11-166D19.1**	ENSG00000255248	EPHA3, SRPX, AEBP1, PCDHB12, CPXM2, OMD, EMILIN1, COL6A2, IGFBP7, SVEP1, MFAP4, TNXB	25
**XLOC_014515**	-	AMBP, STAB2, GNE, AZGP1, BMP10, DSCAM, COL18A1	4
XLOC_015969	-	GP1BA, DPT, MMRN1	1
**XLOC_055355**	-	ITGA2B, COL19A1, LAMA1, ADAM2, IGSF11, NCAM2	25

1. Cell adhesion gene list were derived from gene ontology annotation (GO:0007155) (http://geneontology.org/);
2. lncRNAs in bold are metastasis-associated recurrently deregulated lncRNAs.

Supplementary Table 3. siRNA sequence used for candidate lncRNAs

lncRNA Name	GENCODE ID	siRNA No.	siRNA sense sequences	siRNA antisense sequences
WDFY3-AS2	ENSG00000180769	siRNA-1	GCCUAGAAAUGGCAUGCAUTT	AUGCAUGCCAUUUCUAGGCTT
		siRNA-2	GCCAUACCCUUGACCAGAATT	UUCUGGUCAAGGGUAUGGCTT
		siRNA-3	GCUGUUUGAAUCAGGAAUATT	UAUUCCUGAUUCAAACAGCTT
RP1-153P14.5	ENSG00000227920	siRNA-1	GCAUGCAUGUUAGCAUGACTT	GUCAUGCUAACAUGCAUGCTT
		siRNA-2	GGGCAUUCCUGAAUGAGUGTT	CACUCAUUCAGGAAUGCCCTT
		siRNA-3	GACAGAGAGGUUGACACAATT	UUGUGUCAACCUCUCUGUCTT
AC096579.7	ENSG00000231486	siRNA-1	CCGGACAAAGGACUUUGCUTT	AGCAAAGUCCUUUGUCCGGTT
		siRNA-2	GGCAAAGAAGCAGCUCUUUTT	AAAGAGCUGCUUCUUUGCCTT
		siRNA-3	GGGAAUCCAGGACAGAUAUTT	AUAUCUGUCCUGGAUUCCCTT
HAND2-AS1	ENSG00000237125	siRNA-1	GCCAAGGUACAUCUCAGAUTT	AUCUGAGAUGUACCUUGGCTT
		siRNA-2	CCUCUUGACUUGCCAGUAUTT	AUACUGGCAAGUCAAGAGGTT
		siRNA-3	GCUGGUACUGUCACUUAUATT	UAUAAGUGACAGUACCAGCTT
LINC01018	ENSG00000250056	siRNA-1	CCAUCAAUAACCUCAUCAATT	UUGAUGAGGUUAUUGAUGGTT
		siRNA-2	GGUGACUGAUUUGCGGCAATT	UUGCCGCAAAUCAGUCACCTT
		siRNA-3	CCUAAUGCCUCUGCAUUAATT	UUAAUGCAGAGGCAUUAGGTT
RP11-731F5.2	ENSG00000253364	siRNA-1	CGCCGGUUUCAAUCCUGUUTT	AACAGGAUUGAAACCGGCGTT
		siRNA-2	GGAAGACAAAUAGCAGCUGTT	CAGCUGCUAUUUGUCUUCCTT
		siRNA-3	GCAAGUCUGCCCACAUGUATT	UACAUGUGGGCAGACUUGCTT
RP11-166D19.1	ENSG00000255248	siRNA-1	CCACCAGCCUACUGAGAUUTT	AAUCUCAGUAGGCUGGUGGTT
		siRNA-2	GCAUAAGUUGACCUUGCUUTT	AAGCAAGGUCAACUUAUGCTT
		siRNA-3	GCCCUUUCCAUCAAGAUAGTT	CUAUCUUGAUGGAAAGGGCTT
XLOC_014515	XLOC_014515	siRNA-1	CCAUGGACACAGAAACCAATT	UUGGUUUCUGUGUCCAUGGTT
		siRNA-2	GGAGGUCUUCUACUUAGAUTT	AUCUAAGUAGAAGACCUCCTT
		siRNA-3	CCAGGCACGUUGCACUAUUTT	AAUAGUGCAACGUGCCUGGTT
XLOC_015969	XLOC_015969	siRNA-1	GGAAAGACCUGGGAGUCUUTT	AAGACUCCCAGGUCUUUCCTT
		siRNA-2	GCACUCAAAUCGGCCCUUUTT	AAAGGGCCGAUUUGAGUGCTT
		siRNA-3	GCCAGUUCUCGCAUGAGAUTT	AUCUCAUGCGAGAACUGGCTT
XLOC_055355	XLOC_055355	siRNA-1	GCACGCAAGCAUAAUAUAATT	UUAUAUUAUGCUUGCGUGCTT
		siRNA-2	GCAAGAACUUAUGGGCUUATT	UAAGCCCAUAAGUUCUUGCTT
		siRNA-3	GCAUUCUUCUUAACCACAATT	UUGUGGUUAAGAAGAAUGCTT
Negative control			UUCUCCGAACGUGUCACGUTT	ACGUGACACGUUCGGAGAATT

Supplementary Table 4. qPCR primer sequences used for candidate lncRNAs

lncRNA Name	GENCODE ID	Forward/Reverse	Sequence	Tm (℃)	Product size (bp)
WDFY3-AS2	ENSG00000180769	Forward primer	CTCACCCTCCTCAGTCCCA	61.2	116
		Reverse primer	CGTTTCTCCATCGCCTCTG	61.9	
RP1-153P14.5	ENSG00000227920	Forward primer	CACTGGGCTACACAACGGACA	62.6	116
		Reverse primer	CCCCAATTTACTGACAAGGCAAC	60.3	
AC096579.7	ENSG00000231486	Forward primer	TTCTTTCTGCTTTAGCTTGACT	55.5	138
		Reverse primer	AATCAGGTTCCAAGCACCA	59.1	
HAND2-AS1	ENSG00000237125	Forward primer	CTGTAAACTCTGACTTGCTG	51	75
		Reverse primer	TAATAACGGTTAAGTTGCCAT	54.2	
LINC01018	ENSG00000250056	Forward primer	TATCGGCCATTTGTATGTCCT	59.3	132
		Reverse primer	TGTTAGACACCCCACATCCAT	60.1	
RP11-731F5.2	ENSG00000253364	Forward primer	TTCTCCATCCCAGCTCGCCTT	63.6	163
		Reverse primer	GTGTGAACTCCACGCTGCAA	61.4	
RP11-166D19.1	ENSG00000255248	Forward primer	CACTTCATGCTGCTATGTCG	58	198
		Reverse primer	TCTTCTCAGGCACAATCCTC	57.9	
XLOC_014515	XLOC_014515	Forward primer	CCTGGGTCCTATTACGTGCCAA	65.2	82
		Reverse primer	CGCCTGTCTTCCAGATGCT	61.5	
XLOC_015969	XLOC_015969	Forward primer	CACAGCCACTCACCACATGC	63.9	127
		Reverse primer	TCGGGATCTCTCTGCTCTCACC	65.4	
XLOC_055355	XLOC_055355	Forward primer	CAAAATCAGACCGTAAGGGA	57.7	105
		Reverse primer	TCACCCCTTTCATCTAAGCC	59.1	

Supplementary Table 5. Transwell migration assays in all three liver cancer cell lines for candidate lncRNAs

lncRNA		siRNA No.	#Migration cell								
			HepG2			SMMC-7721			HCCLM9		
**Negative control**		-	548	510	522	281	299	328	385	362	321
**WDFY3-AS2**	**ENSG00000180769**	siRNA-1	352	348	426	187	195	223	390	334	409
		siRNA-2	304	347	308	206	219	222	417	398	435
		siRNA-3	334	351	379	223	201	221	357	429	409
**RP1-153P14.5**	**ENSG00000227920**	siRNA-1	557	628	598	191	186	230	331	356	359
		siRNA-2	626	647	558	229	258	236	351	366	379
		siRNA-3	558	564	527	234	256	225	365	334	354
**AC096579.7**	**ENSG00000231486**	siRNA-1	501	487	463	279	305	340	383	337	338
		siRNA-2	457	493	496	272	263	317	366	364	339
		siRNA-3	495	499	463	292	316	263	295	325	313
**HAND2-AS1**	**ENSG00000237125**	siRNA-1	578	513	580	167	148	183	179	186	207
		siRNA-2	491	508	563	188	203	189	166	177	190
		siRNA-3	554	512	583	146	150	206	165	151	166
**LINC01018**	**ENSG00000250056**	siRNA-1	434	501	455	227	254	309	330	329	272
		siRNA-2	499	522	512	292	307	235	297	324	339
		siRNA-3	588	536	558	239	283	253	291	340	389
**RP11-731F5.2**	**ENSG00000253364**	siRNA-1	602	643	546	331	344	353	314	281	245
		siRNA-2	599	606	544	335	327	331	318	319	342
		siRNA-3	594	585	512	292	273	336	342	331	296
**RP11-166D19.1**	**ENSG00000255248**	siRNA-1	624	606	564	245	261	255	426	433	443
		siRNA-2	705	686	765	241	311	316	439	426	409
		siRNA-3	596	656	645	260	267	291	441	430	398
**XLOC_014515**	**XLOC_014515**	siRNA-1	495	468	516	375	325	385	398	433	437
		siRNA-2	493	496	473	356	343	316	405	428	468
		siRNA-3	459	504	486	295	289	351	406	414	451
**XLOC_015969**	**XLOC_015969**	siRNA-1	544	509	605	289	273	306	445	419	417
		siRNA-2	558	531	485	261	293	295	477	414	475
		siRNA-3	564	515	528	257	329	337	441	478	499
**XLOC_055355**	**XLOC_055355**	siRNA-1	450	487	506	310	322	300	187	186	171
		siRNA-2	480	466	526	337	295	345	195	183	187
		siRNA-3	401	408	530	299	307	280	209	201	182

Supplementary Table 6. CCK8 cell proliferation assays in all three liver cancer cell lines for candidate lncRNAs

lncRNA		siRNA No.	OD450								
			HepG2			SMMC-7721			HCCLM9		
Negative control		-	1.36	1.314	1.345	1.287	1.248	1.282	0.883	0.845	0.866
WDFY3-AS2	ENSG00000180769	siRNA-1	1.48	1.41	1.487	1.139	1.184	1.362	0.907	0.822	0.872
		siRNA-2	1.374	1.383	1.433	1.47	1.319	1.385	0.844	0.991	0.904
		siRNA-3	1.347	1.319	1.319	1.215	1.395	1.401	0.982	0.84	0.927
RP1-153P14.5	ENSG00000227920	siRNA-1	1.398	1.383	1.312	1.33	1.477	1.217	0.971	0.901	0.918
		siRNA-2	1.458	1.365	1.336	1.33	1.263	1.326	0.878	0.8	0.899
		siRNA-3	1.234	1.226	1.433	1.39	1.168	1.261	0.802	0.966	0.912
AC096579.7	ENSG00000231486	siRNA-1	1.351	1.274	1.25	1.305	1.387	1.438	0.924	0.903	0.931
		siRNA-2	1.29	1.268	1.185	1.222	1.375	1.228	0.883	0.859	0.893
		siRNA-3	1.51	1.245	1.267	1.367	1.23	1.266	0.954	0.91	0.907
HAND2-AS1	ENSG00000237125	siRNA-1	1.423	1.442	1.338	1.372	1.363	1.136	0.912	0.846	0.842
		siRNA-2	1.254	1.341	1.396	1.216	1.38	1.106	0.881	0.814	0.86
		siRNA-3	1.472	1.481	1.399	1.293	1.371	1.368	0.859	0.985	0.916
LINC01018	ENSG00000250056	siRNA-1	1.539	1.323	1.306	1.04	1.319	1.143	0.907	0.932	0.935
		siRNA-2	1.445	1.39	1.464	1.377	1.087	1.398	0.884	0.907	0.876
		siRNA-3	1.401	1.559	1.382	1.217	1.417	1.239	0.87	0.821	0.898
RP11-731F5.2	ENSG00000253364	siRNA-1	1.43	1.503	1.207	1.405	1.215	1.306	0.901	0.887	0.904
		siRNA-2	1.503	1.419	1.456	1.198	1.326	1.259	0.854	0.92	0.946
		siRNA-3	1.355	1.521	1.448	1.368	1.229	1.285	0.866	0.952	0.873
RP11-166D19.1	ENSG00000255248	siRNA-1	1.383	1.263	1.432	1.408	1.116	1.125	0.845	0.936	0.825
		siRNA-2	1.346	1.259	1.369	1.204	1.226	1.31	0.926	0.917	0.949
		siRNA-3	1.447	1.465	1.434	1.271	1.36	1.134	0.958	0.937	0.92
XLOC_014515	XLOC_014515	siRNA-1	1.404	1.31	1.366	1.366	1.167	1.157	0.876	0.901	0.914
		siRNA-2	1.305	1.238	1.231	1.207	1.199	1.294	0.861	0.829	0.833
		siRNA-3	1.31	1.503	1.376	1.474	1.252	1.412	0.828	0.836	0.852
XLOC_015969	XLOC_015969	siRNA-1	1.245	1.226	1.197	1.114	1.361	1.204	0.82	0.777	0.839
		siRNA-2	1.226	1.301	1.215	1.478	1.22	1.337	0.857	0.954	0.938
		siRNA-3	1.453	1.426	1.413	1.219	1.217	1.234	0.821	0.87	0.865
XLOC_055355	XLOC_055355	siRNA-1	1.462	1.287	1.378	1.334	1.211	1.341	0.895	0.91	0.924
		siRNA-2	1.261	1.353	1.38	1.212	1.35	1.14	0.944	0.922	0.91
		siRNA-3	1.422	1.51	1.425	1.21	1.272	1.237	0.883	0.871	0.825

Supplementary References

1. Iyer, M.K. et al. The landscape of long noncoding RNAs in the human transcriptome. *Nature Genetics* **47**, 199-208 (2015).
2. Liu, C. et al. NONCODE: an integrated knowledge database of non-coding RNAs. *Nucleic acids research* **33**, D112-D115 (2005).
3. Pruitt, K.D. et al. RefSeq: an update on mammalian reference sequences. *Nucleic Acids Res* **42**, D756-763 (2014).
4. Kelley, D. & Rinn, J. Transposable elements reveal a stem cell-specific class of long noncoding RNAs. *Genome Biology* **13**, 243-253 (2012).
5. The ENCODE Project Consortium An integrated encyclopedia of DNA elements in the human genome. *Nature* **489**, 57-74 (2012).
6. Zhang, H. et al. Comparative transcriptome analysis reveals that the ECM-receptor interaction contributes to the venous metastases of hepatocellular carcinoma. *Cancer Genetics* **208**, 482-491 (2015).
7. Hoshida, Y. et al. Integrative transcriptome analysis reveals common molecular subclasses of human hepatocellular carcinoma. *Cancer Res* **69**, 7385-7392 (2009).
8. Kan, Z. et al. Whole-genome sequencing identifies recurrent mutations in hepatocellular carcinoma. *Genome Res* **23**, 1422-1433 (2013).
